# Survey of transformers and towards ensemble learning using transformers for natural language processing

**DOI:** 10.1186/s40537-023-00842-0

**Published:** 2024-02-04

**Authors:** Hongzhi Zhang, M. Omair Shafiq

**Affiliations:** School of Information Technology, Carleton University, Ottawa, ON Canada

**Keywords:** Transformer model, Natural language tasks, Transformer-based model, Ensemble learning

## Abstract

The transformer model is a famous natural language processing model proposed by Google in 2017. Now, with the extensive development of deep learning, many natural language processing tasks can be solved by deep learning methods. After the BERT model was proposed, many pre-trained models such as the XLNet model, the RoBERTa model, and the ALBERT model were also proposed in the research community. These models perform very well in various natural language processing tasks. In this paper, we describe and compare these well-known models. In addition, we also apply several types of existing and well-known models which are the BERT model, the XLNet model, the RoBERTa model, the GPT2 model, and the ALBERT model to different existing and well-known natural language processing tasks, and analyze each model based on their performance. There are a few papers that comprehensively compare various transformer models. In our paper, we use six types of well-known tasks, such as sentiment analysis, question answering, text generation, text summarization, name entity recognition, and topic modeling tasks to compare the performance of various transformer models. In addition, using the existing models, we also propose ensemble learning models for the different natural language processing tasks. The results show that our ensemble learning models  perform better than a single classifier on specific tasks.

**Graphical Abstract:**

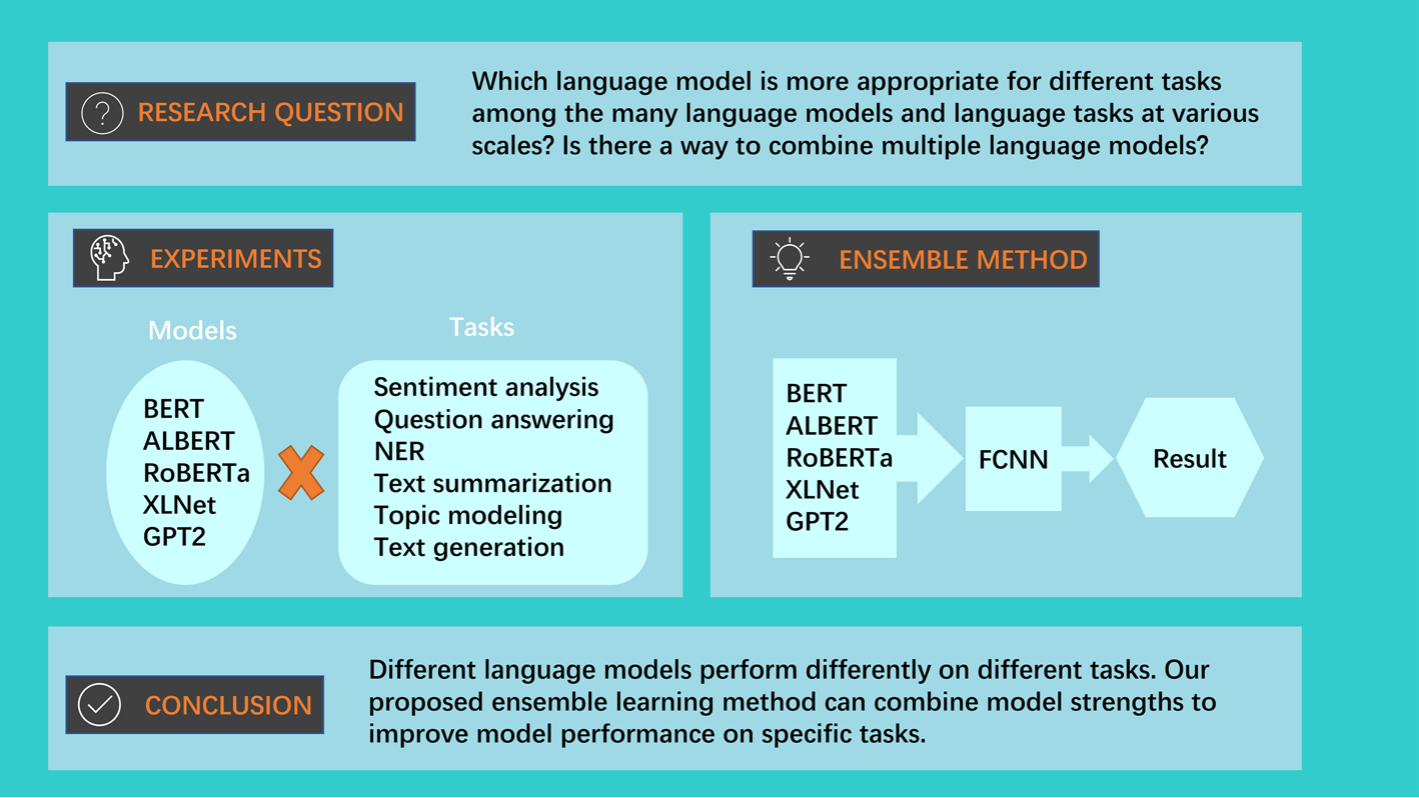

## Introduction

In this section, we introduce the motivation of this research, the main research questions, and the structure of the paper.

### Motivation

Natural language processing (NLP) stands as a technology that facilitates computer-human interaction through the medium of natural languages. It encompasses the artificial processing of human language, empowering computers to not only read but also comprehend it. There are many applications in the field of NLP, covering machine translation, speech recognition, grammar analysis, semantics, and pragmatics. The core of NLP is to segment text corpora for processing, employing tools like ontology dictionaries, word frequency analytics, and contextual semantic scrutiny to isolate the smallest units of meaning.

The key to NLP is to enable natural language communication between humans and computers, where computers not only grasp the meaning of textual language but also express intentions and thoughts in a similar manner. This duality is categorized into ’natural language understanding’ and ’natural language generation,’ forming the two pillars of NLP. However, both domains present formidable challenges. Even in the current theoretical and technological environment, creating a high-quality NLP system remains a challenging long-term goal. However, practical systems with substantial NLP capabilities have emerged for specific applications, some even achieving commercial or industrial success. Examples include multilingual database interfaces, expert systems, machine translation platforms, full-text information retrieval systems, and automatic abstracting tools. The resolution of NLP tasks remains a significant global challenge in today’s context.

The advent of the transformer model in 2017 proposed by Google ushered in a wave of transformer-based models, including BERT, XLNet, and RoBERTa. However, there have not been many studies to comprehensively examine how these models perform in different NLP tasks. Consequently, this paper undertakes the task of comparing five transformer-based models across six distinct NLP tasks through a series of rigorous experiments. Subsequently, we employ the experimental findings to dissect the different performances of these models and delve into the underlying factors contributing to these outcomes. Beyond model comparisons, our objective extends to the integration of these models’ strengths through ensemble learning techniques to yield a more robust and high-performing collective model.

### Research questions

The following are the research questions of this research:

1. What is the role of transformer models towards advanced NLP and text analytics?
2. In different NLP tasks, what are the differences in the performance of different transformer-based models? And why are there these differences?
3. Is there a classification method that can combine the advantages of different models?

### Contributions

This paper has the following main contributions:

1. We have read and organized papers on NLP tasks in the past few years from the literature. In addition, building upon the existing literature, we analyzed the advantages of these papers and what needs to be added.
2. We use five different existing and well-known transformer-based models from the literature to experiment on six types of existing and well-known NLP tasks. We compare the performance of the model based on the experimental results, and analyze the results from the perspective of model structure and training methods.
3. Through analyzing the advantages and disadvantages of different models on different tasks, two ensemble learning models based on the existing models are proposed to be applied to three NLP tasks. Experiments demonstrate that our proposed ensemble learning models outperform a single model based on the transformer model.

### Structure of the paper

In “Introduction” section, we study the research questions and motivations. In “Background” section, we introduced the research background, and described the research task and the model used. In “Review of related works” section, we sorted out the related literature. In “Experimental setup” section, we briefly describe the experimental process and experimental conditions. In “Results and discussion” section, we analyze the results and discuss the reasons. In “Using the ensemble learning methods” section, we proposed an ensemble learning model and conducted experiments on three natural language processing tasks. In “Gap analysis and next steps” section, we analyzed the defects of the article and the next stage of work. In “Conclusions” section we summarize the full text.

## Background

**Fig. 1:**
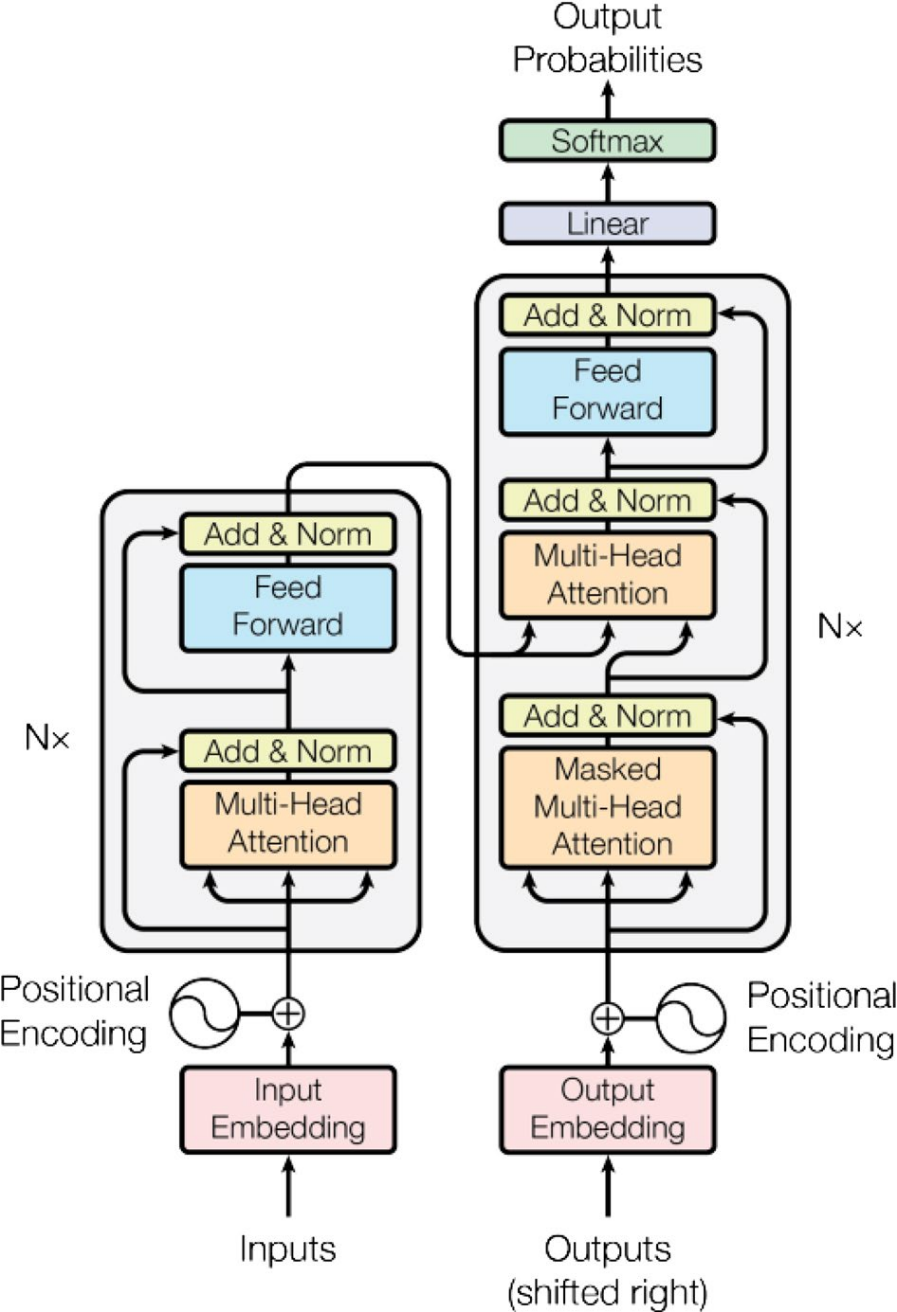
Structure of the well-known transformer model [1]

When it comes to models for solving NLP tasks, many people may think of long short-term memory (LSTM) and other recurrent neural networks (RNNs). But at present, LSTM is becoming less popular in the field of NLP because the parallel computing power of the LSTM model is poor. In addition to that, the transformer model [1] proposed by Google in 2017 has a stronger ability to extract features. In the Stanford Reading Comprehension Dataset (SQuAD2.0) list, the machine’s performance has exceeded human performance, which is largely due to the proposal of the pre-training model BERT which is built based on the encoder of the transformer model. In this paper, we review and compare the different transformer models which are the BERT model, the XLNet model, the RoBERTa model, the GPT2 model, and the ALBERT model. Attention Is All You Need [1] is a paper proposed by Google that takes attention to the extreme. This paper [1] proposed a new model called transformer. This model has the same structure as the seq2seq model and is also an encoder–decoder architecture. Such a structure consists of 6 coding blocks and 6 decoding blocks, as shown in Fig. 1. The encoder maps the text to the middle layer, so the middle layer has a vector form with text information. Then the decoder translates the text information in the middle layer, and many NLP problems can be solved through this process.

### Key natural language processing tasks

In this section, we briefly introduce some of the well-known NLP tasks in the literature.

#### Sentiment analysis

With the growth of the internet, people have become more likely to express themselves online. For example, product reviews on e-commerce sites and what people say on social media about the products and quality of specific brands. These reviews have a huge impact on the product. For instance, brand companies can promptly respond to shifts in public sentiment on social media, especially when negative feedback increases. Sentiment analysis is a core application designed to measure positivity or negativity in text evaluation.

Sentiment analysis is a common task in natural language processing, frequently found in shopping platform reviews. It is the key to product improvement. Through this analysis, businesses gain comprehensive insights into product attributes, enabling improvements across various dimensions.

#### Question answering

Question Answering Systems (QAS) [15] represent an advanced evolution of information retrieval systems. They possess the capability to provide accurate and concise responses in natural language to users’ queries, also expressed in natural language.

Within the area of natural language processing, the QAS stands as a important topic. Its purpose is to address inquiries posed by individuals in natural language form, encompassing a wide array of practical applications. These applications span from intelligent voice interactions and online customer support to knowledge acquisition and empathetic chat services. QAS can be categorized into generative and retrieval-based systems, single-round and multi-round QAS, and those designed for open-ended or domain-specific contexts.

#### Translation

In today’s era of accelerated communication and internet advancements, the exponential growth of information and increasing global interconnectivity have accentuated the challenges of language barriers. Consequently, the demand for machine translation is on the rise. Within the ongoing wave of artificial intelligence, machine translation theory, technology, and future prospects have attracted high interest.

Machine translation involves the transformation of grammatical structures to align with the target language, followed by the translation of individual words from the source language to the target language. This process ensures effective cross-lingual communication in an increasingly interconnected world.

#### Text generation

In NLP, text generation is an important application area. Keyword extraction and text summarization are all applications in the field of text generation. The main techniques of text generation are as follows: synonym-based rewriting method, template-based rewriting method, the rewriting method based on a statistical model and semantic analysis generation model, and neural network-based method.

#### Text summarization

Text summarization tasks revolve around creating a concise and coherent summary that retains the essence and core meaning of key information.

The procedure of utilizing computers to process extensive textual content to generate refined and succinct summaries is what defines text summarization. Summaries serve as efficient means for individuals to grasp a text’s primary content, enhancing both time savings and reading effectiveness. However, manual summarization is labor-intensive and time-consuming, making it inadequate to meet the ever-growing information demands. Hence, the emergence of computer-assisted automatic summarization became imperative. Automatic summarization primarily employs two methods: Extraction and Abstraction.

1. Extraction: Extraction, as an automatic summarization technique, generates summaries by extracting existing keywords and phrases from the source document. These extracted elements form the basis of the summary.
2. Abstraction: Abstraction, on the other hand, takes a generative approach to automatic summarization. It creates summaries by constructing abstract semantic representations and utilizing natural language generation technology to produce coherent summaries.

#### Named entity recognition

Named entity recognition (NER) [2] serves as a fundamental tool across various application domains, including information extraction, QAS, and machine translation. In essence, NER is tasked with identifying three primary categories of textual entities: entities, temporal expressions, and numerical values.

The NER system excels at extracting these entities from unstructured text. And it can be extended to cover a wide range of entity types, such as product name, model number, price, etc., based on specific business requirements. Therefore, the concept of "entity" is broadly defined to include any text fragment that is relevant to a business need, and the goal of NER is to extract these specific entities from the text. In order to achieve this, NER systems typically use both rule-based and model-based approaches.

1. Rule-based methods: Rule-based approaches offer a straightforward means of entity extraction. They are particularly effective for entities with distinct contextual cues or entities with features defined in the text.
2. Model-based methods: From a modeling perspective, named entity recognition constitutes a sequence labeling task. In this case, the input to the model is a sequence containing various elements, such as text and time expressions. The model’s output is also a sequence, with each unit in the input sequence assigned a specific label corresponding to its entity type.

In summary, NER plays a key role in the identification and extraction of relevant textual entities. It is a generalized approach adapted to specific business needs.

#### Topic modeling

Topic modeling is a key technique for identifying topics, and central concepts in a given text.

At its core, topic modeling is a statistical approach that uncovers abstract topics by analyzing a collection of documents. It operates under the premise that if an article touches upon a specific topic, certain distinctive words related to that topic will recur frequently within the text. Typically, an article encompasses multiple topics, each with varying proportions.

From a structural perspective, topic modeling offers a method to reveal underlying themes in textual content. Each topic manifests as a probability distribution over words in the vocabulary. This framework, known as a topic model, assumes a generative role. In other words, it assumes that each word within an article arises from a dual probability selection process: one for choosing a topic and another for selecting a word from the distribution of that topic.

In summary, topic modeling empowers us to uncover the underlying structure of textual data by identifying and characterizing distinct themes, providing valuable insights into the content and main ideas contained within.

### Machine learning and deep learning approaches for NLP before transformer

In this section, we describe some of the well-known machine learning and deep learning methods for NLP tasks in the literature.

#### Rule-based methods

Establishing systems for vocabulary analysis, syntactic and semantic interpretation, question-answering, chatbots, and machine translation predominantly relies on rule-based frameworks. This approach leverages human introspective knowledge, does not require heavy reliance on data, and facilitates rapid system deployment. However, it is not without its drawbacks, notably in terms of limited robustness and generalization capabilities.

The rule-based methodology in NLP often involves abstracting extensive sets of sentences, tailored for specific human-computer interactions, into rules via grammar productions. These rules incorporate key information markers. Subsequently, the system can employ finite state automata generated from these rule sets to convert linguistic input into a parameter sequence. This sequence then guides the corresponding information processing methods. This approach not only enhances the efficiency of natural language understanding but also underscores the rule set’s scalability.

A NLP system, rooted in grammar rule matching, mainly focuses on the transformation of natural language input into machine-understandable parameter data. It achieves its functions primarily through three core modules: word segmentation, parameter labeling, and grammar rule matching.

#### Methods based on machine learning

The concept underlying machine learning-based approaches involves harnessing annotated data to construct a machine learning system, predicated on manually defined features. This system employs learning techniques to determine its parameters, which are then utilized during runtime for data processing and output generation. Notable successes in employing statistical methods have been witnessed in applications such as machine translation and search engines.

NLP tasks encompass a multitude of subtasks within its purview. Traditional machine learning methodologies, including support vector machines (SVM), Markov models, conditional random fields (CRF), and others, have been effectively employed to address these subtasks. Nonetheless, practical applications reveal certain limitations:

1. Dependency on training set quality: Traditional machine learning models heavily rely on the quality of their training data. Manual labeling of the training set is a requisite, which can undermine training efficiency.
2. Field-specific variations: Traditional machine learning models may exhibit varying performance across different domains, weakening their adaptability and underscoring the limitations of a singular learning approach. Creating a training dataset that suits multiple domains requires significant human resources for manual labeling.
3. Complex language features: When faced with high-level, abstract natural language, traditional machine learning struggles to manually label these intricate language characteristics. Consequently, it is limited to learning predefined rules, unable to capture nuanced language features beyond established rules.

#### Methods based on deep learning

Deep learning models are increasingly applied to NLP tasks, utilizing architectures like Convolutional Neural Networks (CNNs) and RNNs.

When applying a fully connected network to NLP tasks, several challenges arise:

1. Variable input length: Different input samples can have varying input and output lengths, making it impossible to fix the number of neurons in the input and output layers.
2. Inability to share features: Features learned from different positions in the input text cannot be shared, leading to inefficiencies.
3. Model complexity: The model tends to have a large number of parameters and requires extensive computations.

To address these issues, RNNs come into play. RNNs scan input data, enabling parameter sharing across all time steps. At each time step, they not only receive input from the current moment but also incorporate information from the previous step, allowing past information to influence current decisions effectively.

Traditional RNNs, however, face limitations. They tend to simply pass along all learned knowledge to the next time step without any processing. Consequently, early knowledge may be overwritten by more recent information, and long-range dependencies are challenging to capture.

LSTM models introduce a gating mechanism to mitigate the vanishing gradient problem in training with long sequences.

By learning word embeddings, deep learning enables the completion of natural language classification and understanding. Compared to traditional machine learning, deep learning-based NLP offers several advantages:

1. Continuous learning: Deep learning continuously learns language features based on word or sentence vectorization, grasping higher-level and more abstract language features to accommodate a broad range of NLP tasks.
2. Automatic feature learning: Deep learning eliminates the need for manual definition of training sets by automatically acquiring high-level features through neural networks.

### Different models based on transformer

In this section, we introduce several models based on the transformer model available in the literature.

#### BERT [3]

Google AI’s introduction of the Bidirectional Encoder Representations from Transformers (BERT) model in 2018 sent shockwaves through the NLP industry, heralding a milestone in the field’s evolution. Notably, BERT exhibited exceptional performance on reading comprehension datasets, but its impact extended far beyond. What set BERT apart was its capacity to concurrently fine-tune contextual representations across different layers, distinguishing it from contemporaneous language models. This unique feature rendered the pre-trained BERT model a versatile tool, well-suited for addressing intricate NLP tasks, often necessitating only minor structural adjustments for tasks like sentiment analysis and question answering.

BERT’s training regimen comprises two main phases: pre-training and fine-tuning. During the pre-training phase, the model undergoes training on diverse unlabeled data, engaging in various pre-training tasks. The subsequent fine-tuning phase initializes the pre-trained BERT model and updates its parameters using task-specific datasets. Despite their shared architectural foundation, fine-tuned BERT models exhibit distinct parameterizations, underscoring their individuality. This nuanced difference between pre-trained and final models stands as a hallmark of the BERT framework.

#### GPT2 [4]

OpenAI introduced the Generative Pre-trained Transformer (GPT) model in their paper titled “Improving Language Understanding by Generative Pre-Training.” Following this milestone, OpenAI also presented the GPT-2 model in their paper titled “Language Models are Unsupervised Multitask Learners.” These models have significantly contributed to the field of natural language processing and have garnered substantial attention for their capabilities in language understanding and generation. The structure of the GPT-2 and the GPT is not much different, but GPT-2 uses a larger dataset for experiments. GPT-2 has a very large amount of training data. BERT, which has attracted widespread attention, used 300 million parameters for training and refreshed 11 NLP records. The GPT-2 launched by OpenAI has as many as 1.5 billion parameters. It is trained on an 8 million web page dataset and covers a wide variety of topics. In the deep learning method, The BERT and GPT-2 models both use transformer technology. The difference is that BERT uses a two-way language model for pre-training, while GPT2.0 uses an earlier one-way language model. Therefore, the types of architectures that GPT-2 can use in pre-training are therefore restricted and cannot fully integrate context.

#### XLNet [5]

**Fig. 2:**
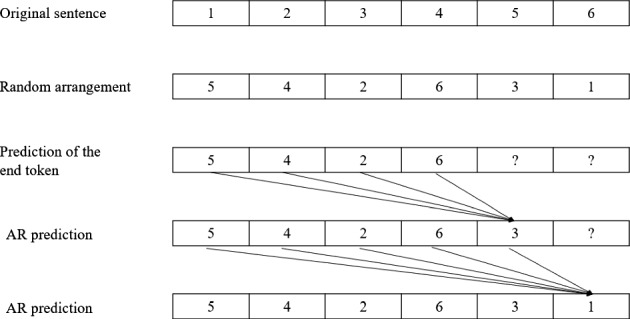
Process of XLNet [5]

The XLNet paper first put forward a point of view, dividing the current pre-training model into two types: Auto Regression (AR) and Auto Encoder (AE). GPT is an AR method that continuously uses the information currently obtained to predict the next output. The BERT model is an AE method that masks some words in the input sentence and then restores the data through the BERT model. This process is similar to a denoising autoencoder. XLNet combines the advantages of the AR and AE methods, and permutation language model (PLM) is used to achieve this goal. The XLNet model can shuffle the order of the tags in the model, and then AR is used for prediction. By using this method, when predicting the token, the two-way information of the token can be used at the same time, and the dependence between tokens can be learned, as shown in Fig. 2.

In order to realize PLM, XLNet proposed Two-Stream Self-Attention and Partial Prediction. In addition, XLNet also uses the Segment Recurrence Mechanism and Relative Positional Encoding in Transformer-XL.

#### RoBERTa [6]

After the XLNet model outperformed the BERT model in NLP tasks, Facebook proposed the a Robustly Optimized BERT Pretraining Approach model (RoBERTa). Compared with the BERT model, the RoBERTa model does not have too many structural differences, but the methods in the pre-training phase have changed. Compared with the BERT model, the RoBERTa model has more model parameters, a larger batch size, and more training data. In addition, the RoBERTa model also has different pre-training methods. First, it deletes the next sentence prediction (NSP) task. Second, it uses dynamic masks. The BERT model gets a static mask during data preprocessing. The RoBERTa model uses dynamic masks: different mask modes are applied to different data sequences. Through this method, different masking methods can be learned by the RoBERTa model for different language representations after a large amount of data training.

#### ALBERT [7]

That the size of a model can have an impact on its performance is a lesson learned from the ongoing advances in language representation learning. Surprisingly, experiments have shown that merely augmenting the number of hidden layers in a model does not necessarily improve performance. To address these challenges, Google researchers proposed a lightweight variant of BERT known as ALBERT, boasting significantly fewer parameters than the original BERT model.

ALBERT accomplishes parameter reduction through two distinctive strategies. Firstly, it undertakes factorization of the embedding parameterization. The model’s objective is to increase the number of hidden layers without expanding the parameter count for word embedding. To achieve this, ALBERT decomposes the extensive word embedding matrix into two smaller matrices, effectively decoupling the hidden layer size from the word embedding size. Secondly, ALBERT introduces parameter sharing across different layers, a technique that reduces parameter spreading as the depth of the network increases. By applying these methods, the model achieves a reduction in parameters while exerting the least possible impact on performance.

### Word vectors in transformer models

Word vectors, commonly referred to as word embeddings, are the foundation of NLP. These embeddings provide a dense vector representation for words, capturing semantic relationships and nuances in meaning. For instance, words with similar meanings tend to be closer in the vector space, enabling models to understand semantic similarities and differences between words.

In the context of transformer models, word vectors play a pivotal role. The initial input to transformer models is the word embeddings of the text. These embeddings are then processed through multiple self-attention mechanisms and feed-forward networks present in the transformer architecture [1]. As the information flows through the layers of a transformer, the model refines these embeddings, capturing contextual information from surrounding words. This ability to understand context is a significant advancement over traditional word embeddings, which are static and lack contextual awareness.

The application of word vectors in transformer models, such as BERT, has led to breakthroughs in various NLP tasks [3]. For instance, models like BERT utilize these embeddings to understand the context around a word, enabling superior performance in tasks like question answering, sentiment analysis, and more. The dynamic nature of transformers, combined with the foundational knowledge captured in word vectors, has made them the state-of-the-art choice for many NLP applications.

### What makes transformer model better for NLP tasks

In this section, we introduced several special mechanisms in the transformer model.

#### Self-attention [1]

There are many similarities between self-attention and attention, but the transformer model uses self-attention to understand and translate other related words into a translation method of the word we are dealing with. Let us look at an example: a wolf does not want to eat a rabbit because it is too thin. Does it represent a wolf or a rabbit? It can be easily judged for us. But for the machine, it is difficult to judge. The Self-attention mechanism can make the machine associate it with the rabbit.

First, the self-attention mechanism will calculate three new vectors, which are query, key, and value. Each query key will make a dot product calculation process. Then use SoftMax to normalize them. Finally, it will be multiplied by value and used as an attention vector. The formula is taken from [1] and is shown in formula 1.

1 $$\begin{aligned} Attention(Q,K,V) = softmax\left( \frac{QK^T}{\sqrt{d_k}}\right) V \end{aligned}$$

#### Multi-head attention [1]

Multi-Head Attention is equivalent to the fusion of several different self-attentions. The transformer uses 8 self-attentions for integration. This can enhance the expressiveness of each layer of attention without changing the number of parameters. And in this way, parallel calculations can be realized, making the calculation more efficient.

#### Positional encoding [1]

Positional Embedding is a very important part in the transformer model. We found that self-attention can extract the dependency relationship between words, but it cannot extract the absolute position or relative position relationship of words. If the order of key and value is disturbed, the result of attention obtained is still the same. In the NLP task, the order between words is very important, so the transformer model uses Positional Embedding to retain word information. Each position in the sequence is assigned a unique numerical identifier, with each number corresponding to a specific vector. These vectors are subsequently added to the word embeddings, thereby incorporating distinct positional information into each word representation.

#### Mask [1]

Mask can mask some values when the parameters are updated, and the masked values will not work during the update. In the transformer model, two types of masks play pivotal roles: padding masks and sequence masks. Since input sequences in a batch can have varying lengths, it’s essential to standardize their lengths. Padding masks serve this purpose by ensuring that input sequences share the same length.

On the other hand, sequence masks are designed to prevent the decoder from accessing future information during the decoding process. In a sequence, at any given time step ’t,’ the output should solely depend on the preceding information up to time ’t.’ Sequence masks are used to conceal information occurring after time ’t,’ ensuring that the decoder remains unaware of future context. This mechanism is integral to the model’s autoregressive nature and its ability to generate output one step at a time, maintaining coherence and adherence to the order of the sequence.

## Review of related works

Financial practitioners often pay attention to economic-related news because they can learn stock trends from this news. For example, the stock price in the past will reflect the past information, and the latest news will participate in the changes in the stock price. Therefore, financial practitioners need to obtain positive or negative information from the latest news on time to make decisions. And people can analyze the information in the news through the sentiment analysis model. However, due to the unavailability of domain-specific languages and large-scale label datasets, financial sentiment analysis is challenging. MISHEV [8] and his team conducted comprehensive research on NLP-based financial sentiment analysis methods. This research covers multiple natural language processing methods, ranging from dictionary-based methods, word and sentence encoders, and transformer models. Compared with other evaluation methods, the transformer shows excellent performance. The text expression method is the main advancement in the accuracy of sentiment analysis. This method inputs the semantics of words and sentences into the model.

Kaliyar et al. [9] studied the bi-directional model BERT. Compared with other word embedding models, BERT is based on the bi-directional idea. It uses a transformer encoder-based architecture to calculate word embedding. Although compared with the BiLSTM model on the transmission encoder, BERT is a powerful feature extractor. But in a larger corpus, BERT has longer training and inference time. It also contains large memory requirements. By designing a fine-tuned BERT model for future research, these practical problems can be alleviated. For small datasets, the performance improvement of BERT will be more noticeable. It shows that the use of pre-trained networks like BERT may be critical to achieving performance in such context-related tasks.

As mentioned earlier, the BERT model has a very good performance in natural language processing tasks. However, in actual tasks, the BERT model requires a lot of computing power. Sun et al. [10] proposed a patient knowledge distillation method, which can compress a large-scale BERT model into a smaller-scale model. Insufficient problems in the calculation of the scale model can be solved by such means. The Patient-KD method introduced in their work achieves multi-layer distillation, enabling the student model to comprehensively absorb the knowledge embedded within the teacher network model. They substantiated the model’s efficacy by subjecting it to a battery of natural language processing tasks, thereby validating its effectiveness and utility.

In the initial phase, pre-training models have gained substantial traction in the realm of natural language processing tasks. However, the extensive adoption of large-scale models has also brought about challenges related to real-time processing and computational constraints. Addressing these concerns, Sanh et al. [11] introduced DistilBERT, an enhanced iteration of the BERT model. DistilBERT features reduced parameters, expedites training, and preserves model performance. Their work demonstrated the viability of training a universal language model through distillation and conducted in-depth analysis of different components via ablation studies.

Bi-directional attention learning can greatly help self-attention networks (SAN), such as the BERT and XLNet models. Song et al. [12] proposed a pre-training scheme “Speech-XLNet” similar to XLNet for unsupervised acoustic model pre-training to learn the voice representation of SAN. The parameters of the pre-trained SAN model were adjusted and updated under the hybrid SAN/HMM framework. They speculate that by shuffling the sequence of speech frames, the permutation in Speech-XLNet can be used as a powerful regularization function to make the SAN model use its attention weight method to focus on the global structure. In addition, the Speech-XLNet model can perform speech representation learning by exploring the context. Various experiments show that Speech-XLNet is better than the XLNet model in training efficiency and performance.

Effectively identifying trends in human emotions in social media can play an important role in maintaining personal emotional health and collective social health. Alshahrani et al. [13] fine-tuned the XLNet model to predict the sentiment of Twitter messages at the personal message level and user level. Since the XLNet model can collectively capture the context and use multi-head attention to calculate the context representation, their method greatly improves the technical level of the benchmark dataset. In addition, using deep consensus algorithms, they can significantly improve accuracy.

Compared with static word embedding, the word embedding method represented by context performs better in many NLP tasks. For example, how is the contextual representation model generated by the BERT model generated? Through research, Ethayarajh et al. [14] learned how words will be represented in a natural context. Initially, their investigation revealed that the uppermost layer of the BERT model and analogous models generated notably more context-specific representations compared to the lower layers. This heightened level of contextual specificity consistently coincided with an increased degree of anisotropy.

Klein and Moin [15] proposed a simple and effective problem generation method. They bundle GPT-2 and BERT and then use an end-to-end trainable approach to promote semi-supervised learning. The problems generated by this method are of high quality and have a higher degree of semantic similarity. In addition, the experiments performed show that the proposed method allows problems to be generated and greatly reduces the burden of complete annotations. The word embedding in a two-way context makes the pre-trained BERT perform better in question answering tasks. In addition, since BLEU and similar scores are weak metrics for evaluating generation ability, they recommend using BertQA as an alternative metric for evaluating the quality of problem generation.

The BERT model performs very well on many NLP tasks and it not only has an English version but also many other voice versions. The study found that the BERT model trained by a single voice is better than the BERT model trained by multiple languages. Therefore, training the BERT model of a specific speech has a great effect on the natural language processing task of a specific language. De Lobel et al. [16] proposed a new Dutch model based on RoBERTa, called Robbert. And through different NLP tasks, it is proved that its performance is better than other language models based on the BERT model. And, they found that Robbert’s model performs better when dealing with smaller datasets.

Chernyavskiy et al. [17] proposed a system specially developed for SemEval-2020 Task 11 in a news article. The model they proposed is based on the architecture of RoBERTa, and then the final model is completed through integrated learning of the model after subtask training.

In work [18], Polignano et al. proposed an Italian language understanding model, ALBERTo. This model is used for training with hundreds of millions of Italian tweets. After training, the model is fine-tuned on a specific Italian task, and the final result is better than other models.

The research of Moradshahi et al. [19] shows that different NLP tasks cannot transfer knowledge through the BERT model. So they proposed HUBERT, a modified version of the BERT model. This model separates the symbols from the roles in the BERT representation by adding a decomposition layer on the BERT model. The HUBERT architecture utilizes tensor product notation, where the notation of each word is constructed by binding two separate attributes together. In extensive empirical research, HUBERT has shown continuous improvement in knowledge transfer across various language tasks.

Wu et al. [20] proposed two methods to identify offensive language behaviors in social media. First, they use supervised classification. Second, they use different pre-training methods to generate different data. In addition, they did good preprocessing work, and they translated the emoji into words. Then, they use the BERT model for identification.

Gao et al. [21] study the feature engineering model, based on the related work in the embedded neural network, and try to use the BERT model with deep neural networks. Then, they proposed TD-BERT models with different forms. In different NLP tasks, they compared its performance with other methods. The results show that the TD-BERT model performs best. Experiments show that the complex neural network used to bring good performance through embedding does not match the BERT and incorporating target information into the BERT can stably improve performance.

In this work, González-Carvajal et al. [22] compared the BERT model with traditional machine learning methods in many aspects. The traditional machine learning NLP method uses the TF-IDF algorithm to train the model. The article compares and analyzes the text analysis experiments of the four methods. In all these classifications, we use two different classifiers: BERT and traditional classifiers.

Baruah et al. [23] use classifiers based on BERT, RoBERTa, and SVM to detect aggressiveness in English, Hindi, and Bengali texts. Their SVM classifier performed very well on the test set, with 3 out of 6 tests ranking second in the official results and fourth in the other. However, through more careful analysis, it can be seen that the SVM classifier performs better because the SVM model has a better classification effect. It is found that the BERT-based classifier can better predict minority groups. It was also discovered that their classifier did not correctly handle spelling errors and deliberate spelling errors. FastText word embedding works better when dealing with orthographic changes.

Lee et al. [24] trained the Korean version of the BERT model, KR-BERT, by using a small corpus. Due to the particularity of Korean and the lack of corpus, it is also very important to use the BERT model for language representation. For this reason, they compared different tokenizers and gradually narrowed the minimum range of tokens to build a better vocabulary for their model. After these modifications, the KR-BERT model they proposed can achieve better results even with a small corpus.

In this paper [25], Li et al. compared the BERT and XLNet models, especially from the comparison of the computational characteristics of the two. Through comparison, they found two points. The first is that the two models have similar computational characteristics. The second is that the XLNet model has a relative position encoding function. On modern CPUs, they have 1.2 times the arithmetic operation and 1.5 times the execution time. At this cost, a better benchmark score was obtained.

As multiple geographic locations are involved, the data is inherently multilingual, leading to frequent code-mixing. Sentiment analysis of the code-mixed text can provide insights into popular trends in different regions, but it is a challenge due to the non-trivial nature of inferring the semantics of such data. In this paper [26], the author use the XLNet framework to solve these challenges. They used the available data to fine-tune the pre-trained XLNet model without any other pre-processing mechanisms.

Ekta et al. [27] proposes a method for studying machine reading comprehension. This method uses eye-tracking data for training and studies the connection between visual attention and neural attention. However they show that this connection does not apply to the XLNet model, although XLNet performs best in this difficult task. Their results show that the neural attention strategies learned by different architectures seem to be very different, and the similarity of neural and human attention does not guarantee optimal performance.

Natural speech processing technology has been widely used in real life. But models such as BERT and RoBERTa need to consume a lot of computing resources. Iandola et al. [28] found that the grouped convolution method improves the efficiency of the computer vision network, so they used this technology in SqueezeBERT. Experiments show that its training speed is 4.3 times faster than BERT.

The BERT model has a good performance in several NLP tasks. However, its performance in certain professional fields is limited. Therefore, Chalkidis et al. [29] found that the application of the BERT model in the professional field requires the following steps: use the additional pre-training of the specific domain corpus to adjust the BERT model or pre-train the BERT model from scratch on the specific domain corpus.

Lee et al. [30] uses the BERT model to implement word embedding. Then the text processing is performed by integrating the two-way LSTM model and the attention mechanism. The accuracy of such an integrated method can reach 0.84.

Bashmal et al. [31] also used an ensemble learning method based on the BERT model. After preprocessing Arabic Tweets, they encode emoticons. Then through the integration of the BERT model and an improved BERT model for processing sentences, a high accuracy rate was finally obtained.

The transformer model has achieved great results in many NLP tasks. However, the transformer model has many parameters and requires a lot of space and computing resources. So how to add a smaller and faster model has become a problem. Nagarajan et al. [32] proposed a new method to reduce the size of the transformer model. The approximate calculations to use simple computing resources and reduce the use of some unimportant weights. Doing so allows the model to gain faster speed with only a loss of accuracy.

There are generally two methods of normalization in neural networks, layer normalization and batch normalization. Shen et al. [33] described why the transformer model uses layer normalization. Later, they proposed a power normalization method, which achieved better results.

While the transformer model has demonstrated proficiency in addressing numerous natural language processing challenges, fine-tuning the model remains an intricate endeavor. In their work, Li et al. [34] introduced a visualization framework aimed at providing researchers with an intuitive means of obtaining feedback during parameter adjustments. This framework enhances clarity during the model’s fine-tuning phase by offering researchers a more transparent view of its behavior and performance.

The BERT model based on the transformer model is also applied in the medical field. Electronic health records are often combined with deep learning models to predict patient conditions. Inspired by this, Rasmy et al. [35] proposed the Med-BERT model, a pre-training model trained through patient electronic health record data. In the experiment, it was found that the Med-BERT model has a higher accuracy rate in predicting patients’ clinical tasks.

With the development of the Internet, it has become easier for people to obtain news and information, and there are more and more false information and false news. As a consequence, Schütz et al. [36] harnessed multiple pre-trained transformer-based models for the purpose of identifying fake news. Their empirical findings underscore the robust capability of transformer models in effectively discerning and detecting fake news.

As the Internet continues to evolve, the prevalence of social media platforms has surged, with a substantial portion of content comprising satire. Identifying satirical language poses a unique challenge due to its distinctive nature. In response, Potamias et al. [37] introduced an approach that amalgamates a recurrent neural network and a transformer model to discern satirical language. Empirical results from their study highlight the enhanced performance of their proposed model when applied to the dataset.

The BERT model released by Google is trained on the English corpus. If you want to apply the BERT model to other models, you need to use corpora of other languages to train the model. Souza et al. [38] used the Spanish corpus to train the BERT model and got good results in the test on downstream tasks. They called the trained model BERTimbau.

The BERT model based on the transformer model has a good performance on many NLP tasks. González-Carvajal et al. [39] described why the BERT model performs better than traditional machine learning methods on natural language processing tasks. Describe the superiority of BERT through different experiments.

The BERT model is a pre-trained model based on the transformer model, while the ALBERT model is a lightweight BERT model. Choi et al. [40] compared the BERT model and the ALBERT model, and then proposed an improved version of the BERT and ALBERT model, the Sentence-BERT model, and the Sentence-ALBERT. Through experimental reality, the proposed model has better performance than BERT and ALBERT.

Koutsikakis et al. [41] used Greek predictions to train the BERT model, and obtained a GREEK-BERT model suitable for Greek NLP tasks. And in the task test of natural language processing. They found that the single-language GREEK-BERT model they trained is better than the M-BERT model and XLM-R model that are suitable for multiple languages. In their research, Hall et al. [42] conducted an extensive review of NLP models and their applications in the context of COVID-19 research. Their focus was primarily on transformer-based biomedical pretrained language models (T-BPLMs) and the sentiment analysis related to COVID-19 vaccination. The comprehensive review encompassed an analysis of 27 papers, revealing that T-BPLM BioLinkBERT exhibited strong performance on the BLURB benchmark, which involves the integration of document link knowledge and hyperlinking into the pretraining process. Furthermore, the study delved into sentiment analysis, leveraging various Twitter API tools. These analyses consistently depicted a positive sentiment among the general public regarding vaccination efforts against COVID-19. The paper also thoughtfully outlines the limitations encountered during the research and suggests potential avenues for future investigations aimed at enhancing the utilization of T-BPLMs in various NLP tasks related to the pandemic.

Casola et al. [43] conducted an extensive study on the increasingly popular pre-trained transformers within the NLP community. While these models have showcased remarkable performance across various NLP tasks, their fine-tuning process poses challenges due to a multitude of hyper-parameters. This complexity often complicates model selection and the accurate assessment of experimental outcomes. The authors commence by introducing and detailing five prominent transformer models, along with their typical applications in prior literature, with a keen focus on issues related to reproducibility. One noteworthy observation was the limited reporting of multiple runs, standard deviation, or statistical significance in recent NLP papers. This shortfall could potentially hinder the replicability and reproducibility of research findings. To address these concerns, the authors conducted an extensive array of NLP tasks, systematically comparing the performance of these models under controlled conditions. Their analysis brought to light the profound impact of hyper-parameters and initial seeds on model results, highlighting the models’ relative fragility. In sum, this study underscores the critical importance of transparently reporting experimental details and advocates for more comprehensive and standardized evaluations of pre-trained transformers in the NLP domain.

In a separate vein, Friedman et al. [44] introduce a transformer-based NLP architecture designed to extract qualitative causal relationships from unstructured text. They underscore the significance of capturing diverse causal relations for cognitive systems operating across various domains, ranging from scientific discovery to social science. Their paper presents an innovative joint extraction approach encompassing variables, qualitative causal relationships, qualifiers, magnitudes, and word senses, all of which are instrumental in localizing each extracted node within a comprehensive ontology. The authors demonstrate their approach’s effectiveness by presenting promising outcomes in two distinct use cases involving textual inputs from academic publications, news articles, and social media.

In the realm of actuarial classification and regression tasks, Troxler et al. [45] delve into the utilization of transformer-based models to integrate text data effectively. They offer compelling case studies involving datasets comprising car accident descriptions and concise property insurance claims descriptions. These case studies underscore the potency of transfer learning and the advantages associated with domain-specific pre-training and task-specific fine-tuning. Moreover, the paper explores unsupervised techniques, including extractive question answering and zero-shot classification, shedding light on their potential applications. Overall, the results eloquently demonstrate that transformer models can seamlessly incorporate text features into actuarial tasks with minimal preprocessing and fine-tuning requirements.

Singh and Mahmood [46] offer a comprehensive overview of the current landscape of state-of-the-art NLP models employed across various NLP tasks to achieve optimal performance and efficiency. While acknowledging the remarkable success of NLP models like BERT and GPT in linguistic and semantic tasks, the authors underscore the significant computational costs associated with these models. To mitigate these computational challenges, recent NLP architectures have strategically incorporated techniques such as transfer learning, pruning, quantization, and knowledge distillation. These approaches have enabled the development of more compact model sizes, which, remarkably, yield nearly comparable performance to their larger counterparts. Additionally, the paper delves into the emergence of Knowledge Retrievers, a critical development for efficient knowledge extraction from vast corpora. The authors also explore ongoing research efforts aimed at enhancing inference capabilities for longer input sequences. In sum, this paper provides a comprehensive synthesis of current NLP research, encompassing diverse architectural approaches, a taxonomy of NLP designs, comparative evaluations, and insightful glimpses into the future directions of the field.

In a separate domain, Khare et al. [47] present an innovative application of transformer models for predicting the thermal stability of collagen triple helices directly from their primary amino acid sequences. Their work involves a comparative analysis between a small transformer model trained from scratch and a fine-tuned large pretrained transformer model, ProtBERT. Interestingly, both models achieve comparable R2 values when tested on the dataset. However, the small transformer model stands out by requiring significantly fewer parameters. The authors also validate their models against recently published sequences, revealing that ProtBERT surpasses the performance of the small transformer model. This study marks a pioneering endeavor in demonstrating the utility of transformer models in handling small datasets and predicting specific biophysical properties. It serves as a promising stepping stone for the broader application of transformer models to address various biophysical challenges.

### Discussion

One year after the transformer model was proposed, the BERT model of the encoder part using the transformer model has gradually become familiar and applied to many NLP tasks. Although the BERT model performs well in various NLP tasks, it is computationally intensive and takes a long time. Therefore, paper [10, 11] proposed a method of knowledge distillation to compress the capacity of the model. Two methods are proposed in paper [10], one is the student model learning k layers from the teacher model, and another one is that learning from every k layer from the teacher model. In the methodology described in [11], the approach leverages the shared dimensionality between teacher and student networks. It involves the initialization of the student network from the teacher network by selectively taking one layer out of every two layers in the model.

Not only the encoder part of the transformer is widely used in NLP tasks, but the GPT model of the decoder part based on the transformer model also performs well in NLP tasks. In addition, the RoBERTa model based on the BERT model and the XLNet model, which improves the BERT model, also has good performance. Paper [8, 14, 22, 23] compares several models. Among them, paper [8] has two contributions. The first is the use of models for sentiment analysis of financial news. There has been very little such work before. The second point is to conduct a lot of comparison experiments, using a lot of different text representation methods and machine-learning classifiers for comparison. In paper [14], Geometry of BERT, ELMo, and GPT-2 embeddings are mainly compared. By analyzing the vectors corresponding to words in different layers, we understand the different embedding representations of the three models. In the article [22], the author compared the classification performance of traditional machine learning that uses vocabulary extracted from a TF-IDF model and the BERT model through several experiments. The empirical evidence of the BERT model’s superiority in average NLP problems classical methodologies have been added through four experiments. In the article [23], the author compared BERT, RoBERTa, and SVM models in three languages. Interestingly, the best performing model in this article is SVM, which shows that the performance of traditional machine learning methods can also surpass the transformer model. In this article, we also discovered the importance of data preprocessing, because the spelling of words will cause errors in word embedding, which will lead to incorrect predictions.

Only using a single model such as BERT or XLNet cannot solve some problems, so paper [13, 15, 21] proposed some solutions combining transformer models with other machine learning methods. In paper [13], they used the XLNet model combined with deep consensus for sentiment analysis. This combination can better improve the accuracy of the model. In paper [15], the article studies the generation and answering of questions. They use the GPT-2 model to combine with the BERT model. This combination makes better use of collaborative question generation and question answering. In paper [17], they use RoBERTa as the main model. But at the same time, additional CRF layers were added, and training was performed on two tasks. The results show that this combination is better than just using RoBERTa. In the article [21], the author proposes the TD-BERT model, which is similar to the model in which a fully connected network is added to the BERT network for classification. The difference is that TD-BERT adds a maximum pooling layer after BERT, which allows the classifier to make better use of location information.

The BERT model uses a lot of English corpora for training and has a very large English corpus. But for NLP tasks in other languages, the BERT model is not competent. In paper [16, 18, 24, 26], other language models based on the BERT model have been established. Among them, paper [16] trained a large number of Dutch corpus to obtain a RobBERT model based on Dutch, and paper [18] established an ALBERTo model for Italian NLP tasks. These two models perform very well in the NLP task of the corresponding language. Since Korean is one of the rich languages that use non-Latin alphabets and lack resources, it is also important to capture language-specific linguistic phenomena. In the article [24], the author proposed a KR-BERT model for Korean NLP tasks. This model uses a smaller corpus for training, which makes the training time of the model shorter and more efficient. People use a lot of informal languages when using social media. A lot of code-mixed languages will be produced, such as mixing two languages. Such sentences will be a big obstacle to sentiment analysis. In the article [26], the XLNet model was used to solve such problems, but it did not perform well. I think that for such code-mixed languages, the corpus is no longer working, so you can try to use traditional machine learning methods or use other data preprocessing methods. The attention mechanism is a very important part of the transformer model. Is such an attention mechanism similar to the human attention mechanism? The paper [27] gave the answer. They found that the higher similarity of human attention and performance is significantly related to the LSTM and CNN models, but it is not true for the XLNet model. The XLNet achieved the best performance, which shows that similar to human attention does not guarantee the best performance. It also shows that the machine can only think in a more advanced way.

Iandola et al. [28] with their SqueezeBERT approach and Nagarajan et al. [32] with their use of approximate computing and pruning. The fine-tuning and pre-training of the BERT model for specific tasks or domains include papers such as Chalkidis et al. [29] with their domain-specific pre-training approach and Rasmy et al. [35] with their work on the medical text. The application of the BERT model in various tasks such as sentiment analysis, fake news detection, and satire detection. Lee et al. [30] use of bidirectional LSTM with attention, Bashmal et al. [31] work on Arabic sentiment analysis, Schütz et al. [36] use a transformer-based approach to fake news detection, and Potamias et al. [37] use recurrent and convolutional neural networks for satire detection. The following papers are focused on comparative analysis and model development, including papers such as Souza et al. [38] with their BERTimbau approach for Brazilian Portuguese, González-Carvajal et al. [39] with their comparison of BERT to traditional machine learning models, Choi et al. [40] with their comparative study of BERT variants, and Koutsikakis et al. [41] with their work on GREEK-BERT for Greek NLP tasks.

Hall et al. [42] and Casola et al. [43] review the use of transformer-based biomedical pretrained language models in COVID-19 research and the importance of reporting experimental details and standardization for reproducibility. Friedman et al. [44] present a joint extraction approach to extracting qualitative causal relationships from unstructured text, which has important implications for cognitive systems and graph-based reasoning. Troxler et al. [45] explore the use of transformer-based models to incorporate text data into actuarial classification and regression tasks. Singh and Mahmood [46] provide a comprehensive overview of current NLP research, including different architectures, a taxonomy of NLP designs, comparative evaluations, and future directions in the field. Khare et al. [47] demonstrate the potential of transformer models in predicting the thermal stability of collagen triple helices directly from their primary amino acid sequences.

## Experimental setup

In our experiment, we mainly use six tasks to compare five different models. In this section, we introduce the datasets and various parameters used by the six tasks. Then we provide the results of the five models on these six tasks and explain the results.

### Benchmark datasets

In this part, we briefly introduce the datasets available in the literature used in different tasks.

#### Coronavirus tweets dataset [48] for sentiment analysis task

For the sentiment analysis task, we use the dataset on kaggle website [48]. These data are obtained from Twitter, and then manually labeled and classified. The dataset includes six columns, namely UserName, ScreenName, Location, Tweet At (time to tweet), Original Tweet (content of Tweet), Label (emotional label). Among them, the content in Original Tweet is the unwashed original Tweet. And, Label contains five categories, namely Neutral, Positive, Extremely Positive, Negative, Extremely Negative. Table 1 shows the dataset we used.

**Table 1:** Coronavirus tweets dataset [48]

UserName	ScreenName	Location	TweetAt	OriginalTweet	Sentiment
3799	48751	London	16-03-2020	@MeNyrbie @Phil_Gahan...	Neutral
3800	48752	UK	16-03-2020	advice Talk to your...	Positive
3801	48753	Vagabonds	16-03-2020	Coronavirus Australia:...	Positive
3802	48754		16-03-2020	My food stock is not...	Positive

#### SQuAD 1.1 [49] for question answering task

Stanford Question Answering Dataset (SQuAD) [49] is a reading comprehension dataset. This data set consists of questions asked by researchers in a series of Wikipedia articles. The answer to each question is a piece of text corresponding to the reading article or question, but the question may not be answered. SQuAD2.0 contains 100,000 questions with answers and 50,000 questions without answers. Because our purpose is to compare the performance of different models on question-answering tasks, we choose to use the SQuAD1.1 dataset. The SQuAD1.1 dataset contains more than 500 articles, including more than 100,000 answered questions.

#### Groningen Meaning Bank corpus [50] for named-entity recognition task

NER, also known as named entity recognition, has many applications in real life and is a very important NLP task. The entity recognition dataset we use has a total of 281,837 sentences and a total of 1,354,149 words, including eight entities. The dataset is shown in Table 2.

**Table 2:** GMB dataset [50]

pos	sentence_idx	Word	Tag
NNS	1.0	Thousands	O
IN	1.0	of	O
NNS	1.0	demonstrators	O
VBP	1.0	have	O

#### CNN daily mail dataset [51] for text summarization task

In today’s digital age, the internet produces an ever-increasing volume of information, leading to the challenge of managing vast amounts of textual data. To address this issue, the task of text summarization comes to the forefront as a solution. Text summarization can be categorized into two main types based on the input data: single-document summarization and multi-document summarization. Single-document summarization involves generating concise summaries from individual documents, allowing users to extract key information from a single source. On the other hand, multi-document summarization goes a step further by creating summaries from a collection of documents related to a specific topic. Text summarization provides users with a concise overview of information from multiple sources, and help them understand complex topics.

In the text summarization task, we use the cnn_dailymail dataset [51]. The dataset includes two columns, article and highlights. The article is the main body of the news article, and the highlight is the summary information.

#### Disaster tweets dataset [52] for topic modeling task 

In NLP tasks, we can extract meaning through a series of levels such as words, sentences, paragraphs, and documents. And topic modeling is the best way to understand text in understanding documents. The process of acquiring, recognizing, and extracting these topics from a collection of documents is commonly referred to as topic modeling.

The disaster tweets dataset includes more than 11,000 disaster-related tweets. The dataset contains five columns, namely id, keyword, location, text, and target. We are going to do topic modeling tasks, so we only keep the two columns of keyword and text. The dataset is shown in Table 3.

**Table 3:** Disaster tweets dataset [52]

id	Keyword	Location	Text	Target
0	Ablaze		Communal violence in...	1
1	Ablaze		Telangana: Section 144...	1
2	Ablaze	New York City	Arsonist sets cars...	1
3	Ablaze	Morgantown, WV	Arsonist sets cars...	1

#### Trump 2020 election speech for text generation task [53]

Text generation has a wide range of applications in real life, which is also an important topic in natural language processing tasks. The text generation task is important for some explanatory texts, such as news and reports. Text generation tasks can also be subdivided into different tasks: text summarization and text expansion tasks and so on. We have already introduced the text summarization task before. In this section, we mainly implement the text expansion task.

For this task, we selected speeches delivered by US president Donald Trump in 2020. We mask the last word of each sentence and then use different models to predict the masked words. The dataset contains 494 sentences.

### Testbed

In this part, we introduce the experimental process and the parameters used in the experiment. Our tasks are all running on google colab. The pre-trained models are listed in Table 4.

**Table 4:** The pre-trained models

Model	BERT	XLNet	GPT2	RoBERTa	ALBERT
Pretrained model	Bert-base-uncased	xlnet-base-cased	gpt2	Roberta-base	ALBERT-base-v2

#### Sentiment analysis task

For the sentiment analysis task, we used the Twitter dataset, and the original tweets contained a lot of dirty data, so we cleaned the data first. We removed hyperlinks, html, numbers, people mentioned, and punctuation in the tweets. Then we convert emotional tags, converting text tags into digital tags. Then we send the data into different pre-trained models. The pre-trained models are listed in Table 4. The detailed hyperparameters are listed in Table 5.

**Table 5:** hyperparameters used in different tasks

	Optimizer	Learning rate	Batch size	Epochs
Sentiment analysis	Adam	1e−5	32	5
Question answering	Adam	1e−5	32	5
Name entity recognition	Adam	1e−5	64	3
ext summarization	Adam	1e−5	32	5

#### Question answering task

For the question-and-answer task, we used the SQuAD1.1 dataset. For the BERT, ALBERT, and RoBERTa methods, we used the question answering function in the transformer library. For different models, we load different pre-trained models. The detailed hyperparameters are listed in Table 5.

#### Named-entity recognition task

For the NER task, we mainly use the xxForTokenClassification function in the transformers library, for example, the BertForTokenClassification function for the BERT model. The detailed hyperparameters are listed in Table 5.

#### Text summarization task

The detailed hyperparameters are listed in Table 5. In our study, we leveraged BERT and RoBERTa as encoder–decoder models for text summarization. This approach applies a transformer-based model to map from the input space (encoder: source text) to the output space (decoder: summary text). An instance of EncoderDecoderModel from the transformers library is initialized with the pre-trained model as both the encoder and decoder. Specific tokens and parameters are then set in the config for the model. Then we utilize the Seq2SeqTrainer with a specific set of TrainingArguments for training our model. The model is trained to generate the summary text given the input text.

#### Topic modeling task

In the topic modeling task, we used Twitter with disease topics to conduct experiments. We get a topic after topic modeling for each tweet and compare it with the actual topic label to get the result. In the topic modeling task, we use the BERTopic function to evaluate different models, and the parameters used are listed in Table 6.

**Table 6:** The detailed hyperparameters

Maximum number of topics	The number of keywords selected for each topic
219	5

#### Text generation task

For text generation tasks we use Donald Trump’s campaign speech. For BERT, XLNet, Albert and RoBERTa models, we mask the last word of each sentence, and then predict the mask based on the previous text. For the GPT2 model, we delete the last word of each sentence so that the GPT2 model generates the last word based on the previous text.

## Results and discussion

In this section, we show the experimental results. At the same time, we also analyze the reasons for this result from various angles.

### Sentiment analysis task

In this section, we show the results of different models on sentiment analysis tasks and give the reason.

#### Results

The results of the sentiment analysis task are shown in Table 7.

**Table 7:** Results for sentiment analysis task

Model	BERT	GPT2	XLNet	RoBERTa	ALBERT
Acc	0.76434	0.64691	0.68825	0.71695	0.76645
F1	0.77443	0.65966	0.70266	0.72899	0.77559
P	0.77851	0.67552	0.70387	0.73279	0.78785
R	0.77297	0.65249	0.70604	0.72802	0.76654

**Fig. 3:**
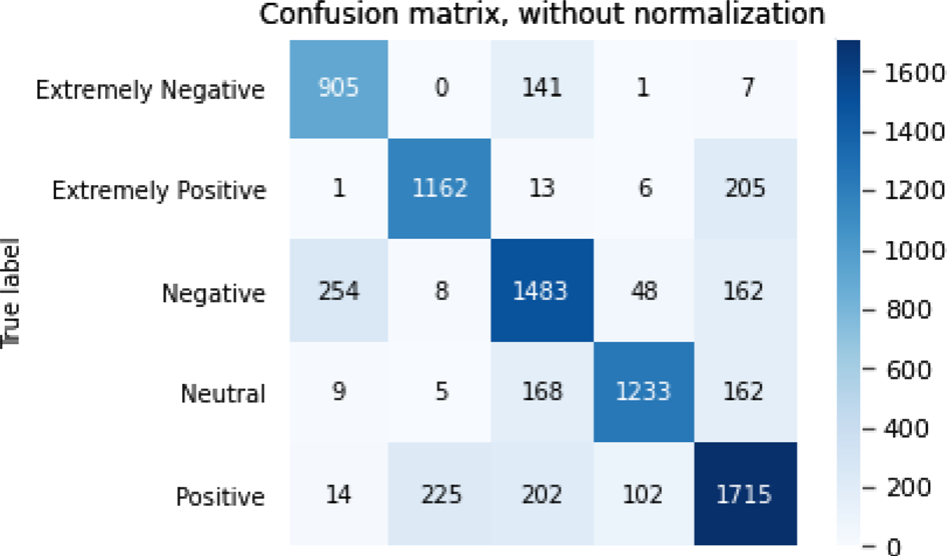
Confusion matrix of BERT

**Fig. 4:**
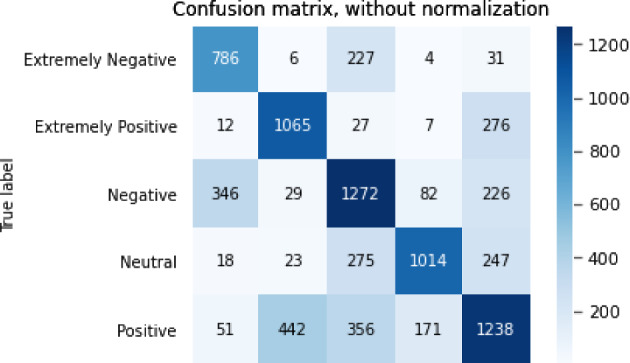
Confusion matrix of GPT2

**Fig. 5:**
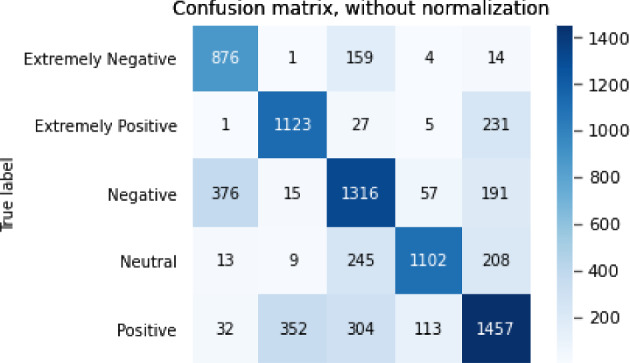
Confusion matrix XLNet

**Fig. 6:**
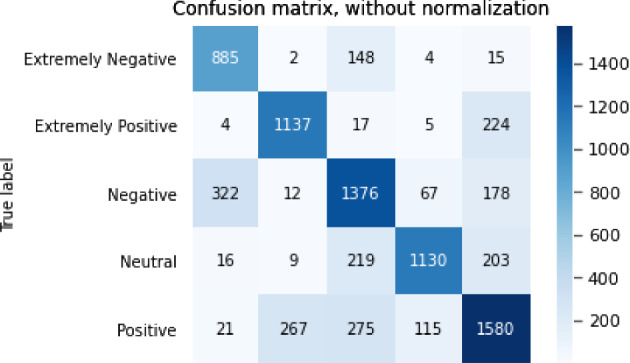
Confusion matrix RoBERTa

**Fig. 7:**
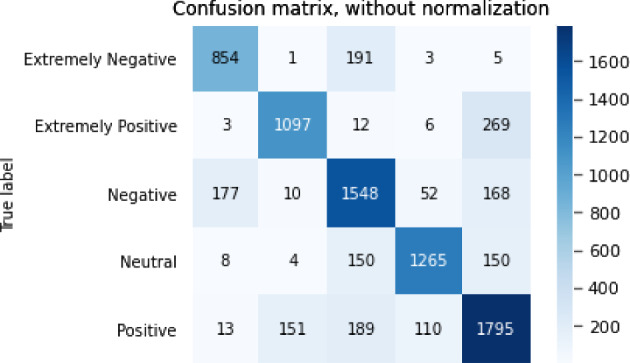
Confusion matrix of ALBERT

The confusion matrix for the sentiment analysis task is shown in Figs. 3, 4, 5, 6,  7.

From the accuracy table and the confusion matrix, we can see that in the sentiment analysis problem, the ALBERT model performed the best. The accuracy of the ALBERT model reached 0.766 and the F1 score reached 0.776. The GPT2 model performed the worst, with an accuracy of only 0.647 and an F1 score of 0.660.

#### Discussion on the results of sentiment analysis task

The GPT-2 model presents unique characteristics that contribute to its relatively suboptimal performance. Our observation from the learning curve denotes that the loss curve of the GPT-2 model’s validation set intersects the training set at the fifth epoch. This suggests that GPT-2 may demand a prolonged training period, incorporating more epochs, to reach its peak performance. Moreover, GPT-2 employs a unidirectional language model in its pre-training stage, restricting it to absorb only prior information. While this is suitable for tasks such as text generation, it falls short for sentiment analysis tasks, which require a comprehensive understanding of the contextual information. Furthermore, GPT-2 lacks a fine-tuning stage for downstream tasks, which, despite its extensive pre-training on large corpora enabling decent performance in various NLP tasks, results in comparatively inferior performance to models incorporating a supervised learning fine-tuning stage.

As the BERT model stands as the second-best performer, we proceed to a comparison between the ALBERT model and BERT. Notably, the ALBERT model diverges from BERT in its training approach. Instead of utilizing the next sentence prediction (NSP) task employed by BERT, the ALBERT model employs the Sentence Order Prediction (SOP) task.

The NSP task in BERT comprises a binary classification task, where positive samples are generated by extracting two consecutive sentences from the same document, while negative samples entail sentences from different documents. This task primarily aims to enhance downstream task performance. However, the NSP task, in essence, encapsulates two subtasks: topic prediction and relationship consistency prediction. Notably, topic prediction is relatively straightforward, focusing on discerning differences in topics between two sentences. On the other hand, relational consistency prediction poses a more complex challenge.

The SOP prediction task, adopted by the ALBERT model, offers unique advantages. By selecting sentences from the same document, this task effectively eliminates any influence of sentence order on the subject matter. Furthermore, the ALBERT model dispenses with the dropout layer, a change that further contributes to its superior performance in downstream tasks. These modifications collectively underscore why the ALBERT model excels, particularly in our sentiment analysis tasks.

### Discussion on the results of question answering task

In this section, we show the results of different models on question-answering tasks and give the reason.

#### Results

The results of the question-answering task are shown in Table 8.

**Table 8:** Results of question answering task

Model	BERT	XLNet	RoBERTa	ALBERT
EM	0.794	0.854	0.886	0.830
F1	0.873	0.921	0.951	0.901

The metric EM(exact match) means the percentage of predictions that match any one of the ground truths answers exactly.

According to the results that are shown in Table 8, the RoBERTa model has the best performance on question-answering tasks and the BERT model is not that good compared to other models.

#### Discussion of question answering result

In our question-answering task experiment, we observed a significant performance contrast between the RoBERTa and BERT models. We analyze and focus on the salient factors that contribute to the RoBERTa model’s superior performance and the relative shortcomings of the BERT model. Firstly, one notable aspect of the RoBERTa model is its expansive use of training data during the pre-training phase. An increased corpus size has been widely recognized in the literature as a highly effective strategy for improving a model’s performance, particularly in terms of generalizability and robustness. Secondly, the RoBERTa model boasts a greater number of parameters compared to the BERT model. This larger parameter space could provide more capacity for RoBERTa to learn intricate representations and patterns from the data, contributing to its superior performance. The third point of differentiation lies in the pre-training tasks. Specifically, RoBERTa omits the NSP task, which has been found to be non-essential, or even potentially counterproductive, in downstream tasks such as question-answering systems. This modification is likely to free up model’s capacity for learning more beneficial representations. The fourth distinction involves the masking strategy. Unlike BERT, which employs a static masking approach during data preprocessing, RoBERTa uses a dynamic mask, generating a fresh mask pattern for each input sequence. This flexibility allows RoBERTa to continuously adapt to different masking strategies as it absorbs large amounts of data, thereby learning varied language representations. For BERT, its static masking strategy and reliance on predicting masked tokens may limit its ability to learn diverse and comprehensive language representations. This limitation may become prominent in question-answering tasks, where understanding context and nuanced language is crucial.

### Named-entity recognition task

In this section, we show the results of different models on named-entity recognition task and show the explanation.

#### Results

The results of the NER task are shown in Table 9.

**Table 9:** Results for NER task

Model	BERT	XLNet	RoBERTa	ALBERT
Acc	0.935	0.954	0.884	0.952
F1	0.986	0.988	0.918	0.990

#### Discussion of named-entity recognition result

The first reason why the ALBERT model performs well is the use of Sentence Oder Prediction (SOP) in the pre-training phase. Compared with the NSP used in the BERT model, SOP performs better in downstream tasks. At the same time, the dropout is removed in the mask stage, which will not only make the memory usage low but also promote the performance of downstream tasks.

Although the XLNet model is an autoregressive language model, it can still introduce contextual information at the same time. This is because the XLNet model uses a permutation language model. The XLNet model randomly arranges context so that context information can be introduced at the same time during the training process, which is not possible with general autoregressive language models. Secondly, the XLNet model is an autoregressive language model and does not have the disadvantages of the self-encoding language model, that is, the error caused by the difference between the fine-tune stage and the pre-training stage. Finally, the XLNet model will perform better on long texts, and the dataset we use is also longer text. So, the XLNet model performs well in the NER task.

The RoBERTa model’s general performance in our NER experiments can be attributed to a few key factors. While RoBERTa’s strengths lie in understanding broad language contexts, NER tasks require more localized pattern recognition. RoBERTa’s extensive pre-training and dynamic masking strategy may not align perfectly with this demand. Furthermore, its larger model capacity might introduce unnecessary complexity, potentially leading to overfitting or dilution of task-specific patterns in NER tasks. Lastly, NER tasks often benefit from architectures that capture sequential text information more effectively than what the transformer-based architecture of RoBERTa might provide. Consequently, RoBERTa’s performance in NER tasks appears to be moderate, suggesting the need for further optimization or model adaptation specific to NER tasks.

### Text summarization task

In this section, we show the results of different models on text summarization tasks and show the explanation.

#### Results

The results of the text summarization task are shown in Table 10.

**Table 10:** Results for text summarization task

Model	BERT	RoBERTa
rouge1	0.1406	0.2864
rougeL	0.1097	0.2306

#### Discussion on the results of text summarization result

In our text summarization task experiment, we observed interesting performance dynamics between the BERT and RoBERTa models. Contrary to our expectations, the BERT model outperformed the RoBERTa model. This observation invites an in-depth analysis of the salient features that may contribute to BERT’s superior performance and the relative shortcomings of the RoBERTa model in text summarization tasks. The RoBERTa model, which was expected to perform optimally due to its extensive training set and large parameter count, yielded only moderate results. RoBERTa’s strength in its extensive pre-training phase should theoretically confer a robust generalization ability. Additionally, its larger parameter count should provide a greater capacity for learning complex patterns in downstream tasks. Moreover, RoBERTa’s dynamic mask allows it to learn diverse language representations, a feature that theoretically should boost its performance in downstream tasks. Despite these advantages, RoBERTa’s performance was not as good as BERT in our text summarization tasks. This could be attributed to a few factors. Firstly, text summarization tasks require a balanced understanding of both global (entire document level) and local (sentence or paragraph level) contextual information. BERT’s bidirectional transformers excel in understanding this balance by encoding each word in the context of its preceding and following words, which could be a major contributing factor to its superior performance. Secondly, text summarization tasks often involve intricate rephrasing, condensation, and selection operations, which require a deep understanding of the source text and a strong semantic understanding ability. BERT’s pre-training tasks, including Masked Language Model (MLM) and NSP, are designed to learn deep bidirectional representations and might be more aligned with the requisites of text summarization tasks compared to RoBERTa’s single MLM pre-training task.

### Topic modeling task

In this section, we show the results of different models on topic modeling tasks and show the explanation.

#### Results

The results of the topic modeling task are shown in Table 11.

**Table 11:** Results for topic modeling task

Model	BERT	XLNet	GPT2	RoBERTa	ALBERT
Accuracy	0.19635	0.15068	0.36986	0.17351	0.36073

We generate topics by using the BERTopic method. Each tweet generates five topics, and the prediction is correct if the labeled topic is included. From the results in the table, we can see that the GPT2 model and the RoBERTa model perform very well. The accuracy of the GPT2 model can reach around 0.37. But the accuracy of the XLNet model is relatively low and only around 0.15.

#### Discussion on the results of topic modeling result

For the topic modeling tasks, we utilized BERTopic, a topic modeling technique that leverages class-based TF-IDF and word embeddings to generate dense explanatory clusters. This process emphasizes keywords in the topic descriptions, thereby augmenting their comprehensibility. As our approach primarily relied on the pre-trained models for word embeddings, the pre-training specifics of these models are particularly relevant.

The GPT-2 model, which demonstrated superior performance, is characterized by a vast corpus used in its pre-training phase, along with a larger parameter set. It employs unsupervised learning during this phase, covering a broad spectrum of topics and enhancing its generalizability across diverse downstream tasks. In our topic modeling task, we bypassed the training phase and directly applied these pre-trained models. This approach inherently favors more versatile models like GPT-2, explaining its exemplary performance.

On the other hand, the XLNet model’s performance was relatively moderate. While XLNet also boasts a large parameter count and employs a permutation-based training strategy to learn a wide range of topics, it might not have been as effective as GPT-2’s left-to-right language modeling approach for our specific task. XLNet’s permutation-based learning could introduce complexity and possibly weaken the localized contextual understanding required for effective topic modeling.

In summary, the superior performance of the GPT-2 model in our topic modeling tasks can be attributed to its broad unsupervised pre-training and greater generalizability. While XLNet is a powerful model, its unique pre-training strategy might not be as well-suited to the specific requirements of topic modeling tasks.

### Text generation task

In this section, we show the results of different models on text generation tasks and show the explanation.

#### Results

From the above table, we can still find that the GPT2 and RoBERTa models have achieved very good performance. The accuracy of the RoBERTa model is around 0.42 and the accuracy of the XLNet model is only about 0.11. The results are shown in Table 12.

**Table 12:** Results for text generation task

Model	BERT	XLNet	GPT2	RoBERTa	ALBERT
Text Generation	0.31578	0.11133	0.36234	0.41700	0.26518

#### Discussion on the results of text generation task

For the text generation tasks, we did not train the models on downstream tasks. Rather, we utilized the existing pre-trained models, which provided an advantage to models like RoBERTa and GPT-2, both of which benefit from a substantial pre-training corpus and unsupervised learning during this phase.

In the case of RoBERTa, our methodology involved masking the last word of each sentence for prediction. RoBERTa, developed with a dynamic masking mechanism during its pre-training phase, generates a unique masking pattern for each input sequence. This leads to enhanced randomness and allows the model to learn a wider variety of patterns, consequently improving its performance in text generation tasks.

In contrast, GPT-2’s superior performance can be ascribed to its architectural alignment with the task’s requirements. Given that we used the first half of each sentence to predict the final word, GPT-2’s unidirectional language modeling, focusing on left-to-right context, gives it a natural advantage over bidirectional models like BERT. Coupled with GPT-2’s extensive pre-training corpus, this structural advantage contributed to its excellent performance.

However, XLNet, despite its large pre-training corpus, displayed only moderate performance. This can be traced back to our task structure. Given that we didn’t employ downstream task tuning but rather direct text generation, GPT-2’s unsupervised learning approach was more suitable. XLNet’s permutation-based training may not be as compatible with such task requirements, which could explain its relative underperformance.

In conclusion, while RoBERTa’s dynamic masking and GPT-2’s unidirectional language modeling and unsupervised learning strategy proved advantageous in our text generation tasks, XLNet’s permutation-based learning might not be as well-suited to the specific task structure. These findings highlight the importance of aligning model selection and task design in NLP experiments.

### Statistical significance of model comparisons

We employed the paired t-test, a statistical method suitable for comparing the means of two related groups. The paired t-test is a statistical test used to determine whether there exists a statistically significant difference between the means of two groups, based on the premise that the variations between paired observations conform to a normal distribution.

For each task, we paired the results of the models being compared. The t-test results indicated that the differences in performance metrics (e.g., accuracy, F1 score) between models were statistically significant at a p-value threshold of 0.05.

The statistical tests reinforce our model comparisons, confirming that the observed differences in performance are not due to random variations but are statistically significant. This lends greater confidence to our evaluations and the subsequent conclusions drawn from them.

### Efficiency analysis

In this section, we analyze the efficiency of each model. Table 13 shows the GPU usage of each model in sentiment analysis tasks.

**Table 13:** GPU usage of different models

Model	BERT	XLNet	GPT2	RoBERTa	ALBERT
GPU/GB	4.47	4.97	3.99	4.30	3.27

## Using the ensemble learning methods

In this section, we propose our ensemble learning models using the existing and well-known ensemble learning [54, 55] and apply these to the natural language processing tasks. Then, we compare the results of the ensemble learning model with the single model and analyze the reasons.

### Using the ensemble learning method for transformers

In real life, when we use machine learning methods, we often only get a classifier that is more accurate in a single category. People want to train to obtain a classifier that is more accurate in each category, which uses an ensemble learning method. Ensemble learning can combine multiple weak classifiers obtained by training so that a classifier with better performance in many aspects can be obtained. Ensemble learning can be divided into three categories as the following:

1. Bagging (reduce variance) [54, 55]
2. Boosting (reduce deviation) [54, 55]
3. Stacking (improve prediction results) [54, 55]

#### Architecture

In our sentiment analysis task, we also integrate different classifiers through ensemble learning to obtain a strong classifier with better performance. We first train several transformer-based language models separately and then integrate these models through neural networks.

#### Procedure

1. The five transformer models are trained separately, and then the model is evaluated through the accuracy of the model and the confusion matrix.
2. The confusion matrix is used to select the best model in the five categories (BERT, XLNet, ALBERT, RoBERTa, GPT2).
3. The outputs of the four models are cascaded and then put into a deep neural network for training.
4. Determine the structure of the deep neural network.
5. Compare the training result of the ensemble model with the training result of the single model.

### Using the ensemble learning method for sentiment analysis task

In this section, we discuss in detail our ensemble learning model for the sentiment analysis task.

#### Customized architecture

**Fig. 8:**
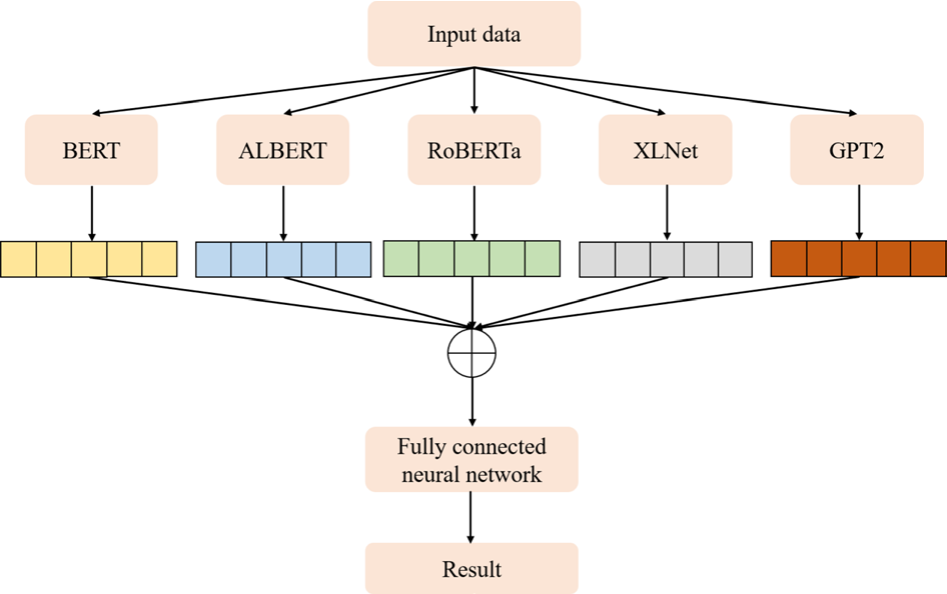
The ensemble learning model for sentiment analysis

The system design for the ensemble method of the sentiment analysis task is shown in Fig. 8. The structure of the deep neural network is shown in Table 14.

**Table 14:** The structure of fully connected neural network

Layer	Number of neurons/dropout rate
Dense	20
Dropout	0.5
Dense	10
Dropout	0.5
Dense	5

For the first layer of the network, we used 20 hidden units. In order to prevent the model from overfitting, we used another dropout layer with a scale of 0.5. Then we added another fully connected layer of 10 hidden units. Then there is a dropout layer with a ratio of 0.5. Finally, we use a fully connected layer of 5 hidden units for classification.

#### Hyperparameter

The detailed hyperparameters are shown in Table 15.

**Table 15:** The detailed hyperparameters

Optimizer	Activation	Dropout ratio
Adam	Softmax	0.5

#### Dataset

Both the training set and the test set used in our experiment are provided by [48].

#### Design discussion

We can see from the confusion matrix and ROC curve that each classifier has different classification effects in different categories. We hope to use ensemble learning methods to combine the advantages of the classifier to improve the accuracy of classification. For multi-classification problems, the model is generally classified by the Softmax function. In order to combine the advantages of different classifiers, we send the output of the five classifiers into the deep neural network, and then for each classifier, we use the same weight. The reason why the Softmax function is not used for classification is that it cannot combine the outputs of different models. The use of deep neural networks can not only combine the outputs of different classifiers but also distribute the weight of each classifier through network calculations to obtain new outputs. We found that the classifier after ensemble learning performed better on the model.

#### Results

**Fig. 9:**
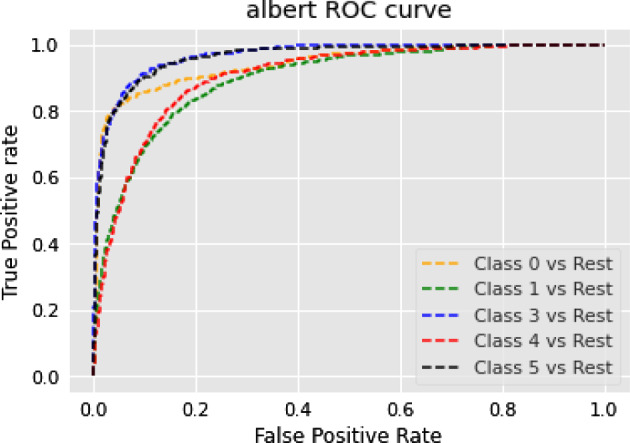
ROC curve for the Albert model

**Fig. 10:**
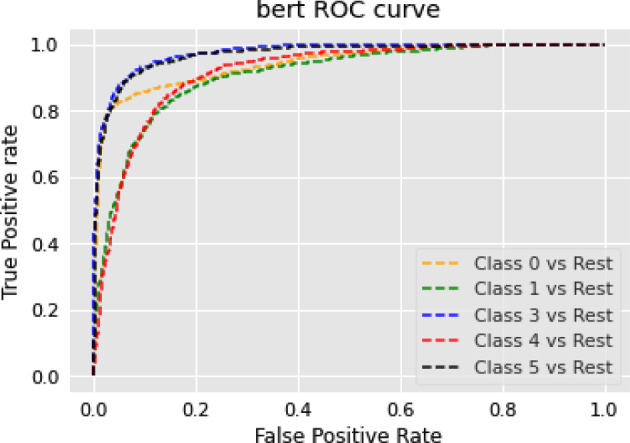
ROC curve for the BERT model

**Fig. 11:**
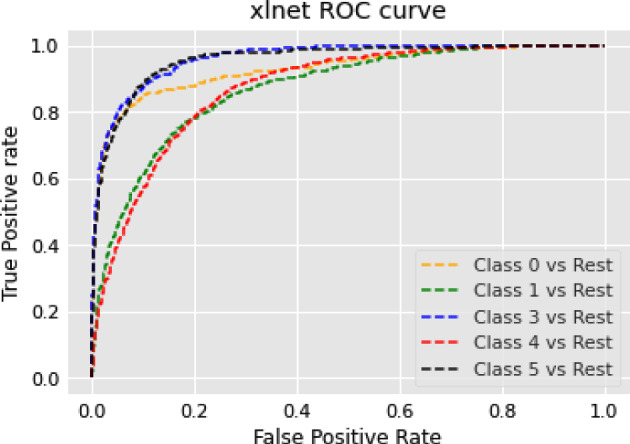
ROC curve for the XLNet model

**Fig. 12:**
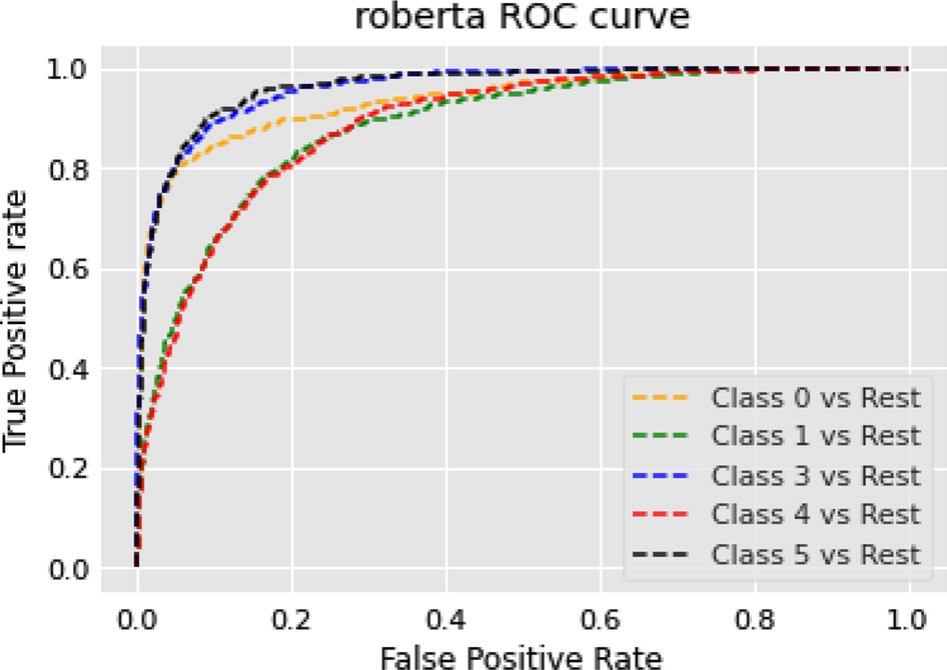
ROC curve for the RoBERTa model

**Fig. 13:**
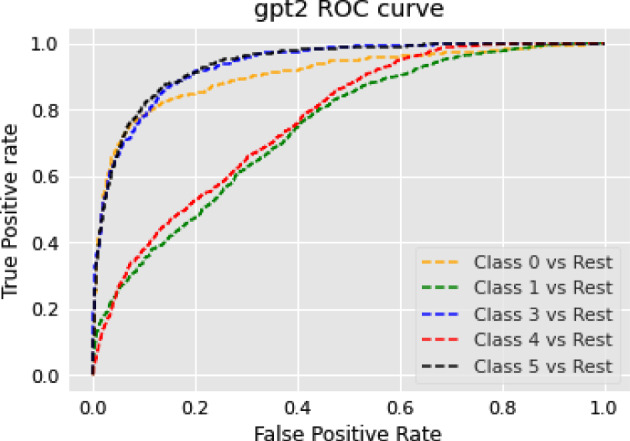
ROC curve for the GPT2 model

**Fig. 14:**
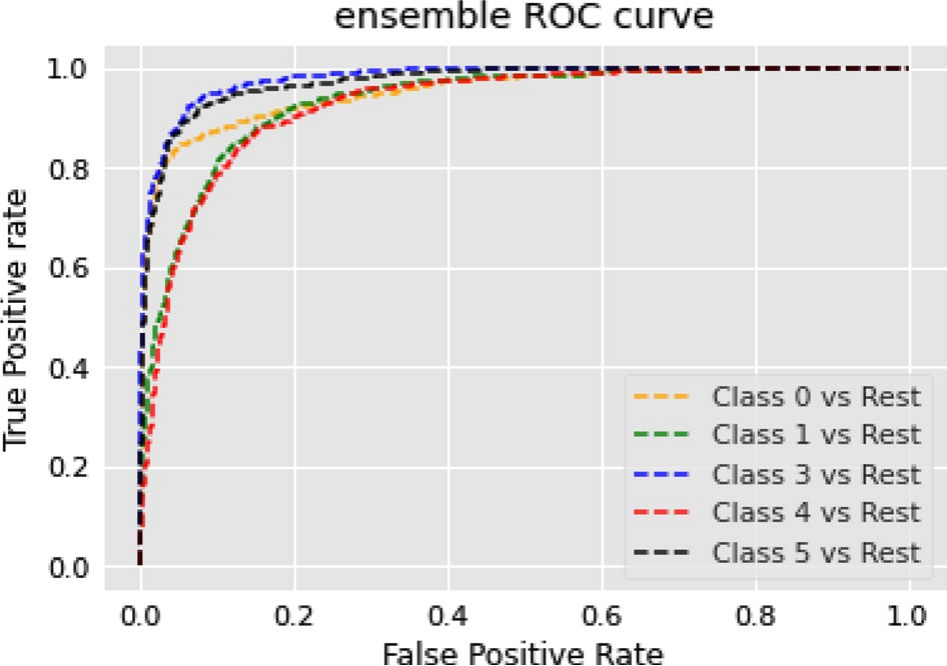
ROC curve for the ensemble learning model

First of all, we can see the results from Figs. 9, 10, 11, 12, 13, 14 and Table 16. The ALBERT model performs best in a single model, with an accuracy of 0.766 and an F1 score of 0.775. But the ensemble learning model we proposed can make the accuracy reach 0.787, and the F1 score can reach 0.795.

**Table 16:** Results for sentiment analysis task

Model	BERT	GPT2	XLNet	RoBERTa	ALBERT	Ensemble learning
Acc	0.764	0.647	0.688	0.717	0.766	0.787
F1	0.774	0.660	0.703	0.729	0.775	0.795
P	0.779	0.676	0.704	0.733	0.788	0.811
R	0.773	0.652	0.706	0.728	0.767	0.786
AUC	0.942	0.859	0.918	0.927	0.935	0.953

After careful inspection of the confusion matrices of the individual models, a clear pattern emerged, with each model exhibiting different performance in different categories. In order to improve the performance of the model on each classification, we chose to use ensemble learning techniques.

Our chosen ensemble method involves channeling the output of trained models into a deep neural network, leveraging the network’s intrinsic characteristics to classify the data effectively. Within the neural network’s hidden layers, the initial layers tend to grasp low-level, elementary features. As the neural network progresses, these basic features are combined to identify more complex patterns and features.

Furthermore, this approach helps to fuse features derived from the output of a single model, enabling the learning of a wider range of features. Finally, such an ensemble learning approach enriches the classification capabilities of our model, thereby improving the overall performance.

### Using the ensemble learning method for NER task

In this section, we discuss in detail our ensemble learning model on the NER task.

#### Customized architecture

**Fig. 15:**
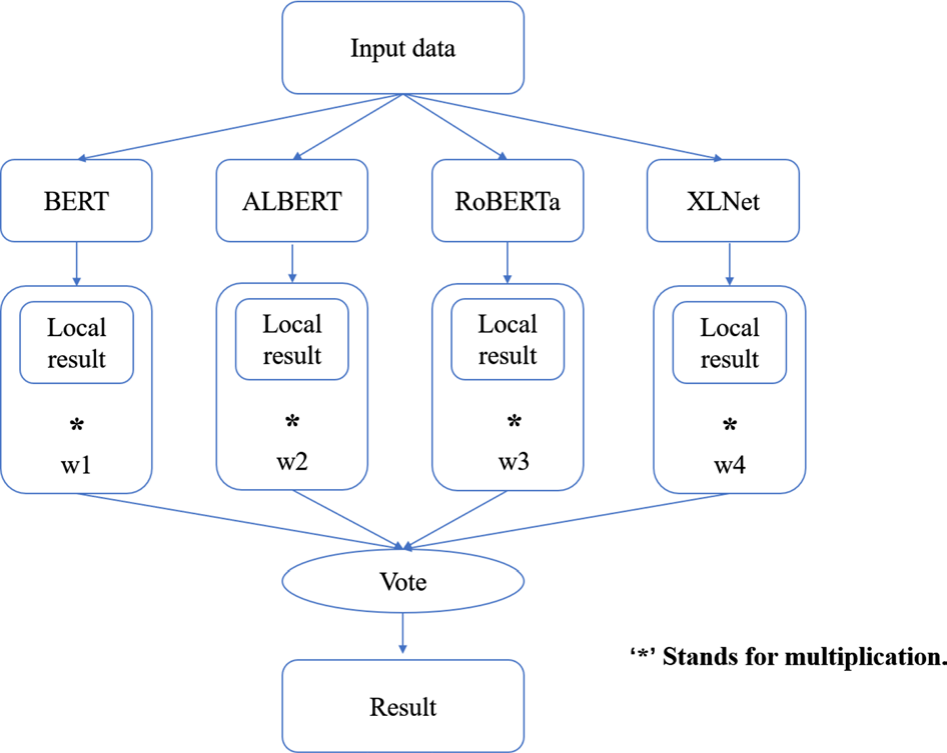
The ensemble learning model for NER task. ‘*’ Stands for multiplication

In the NER task, the system design for the ensemble method is shown in Fig. 15.

#### Hyperparameters

The Hyperparameters are shown in Table 17.

**Table 17:** The weight of different classifiers

Model	w1	w2	w3	w4
Weight	0.938	0.951	0.894	0.964

#### Dataset

The dataset we used in this experiment is provided by [50]. We randomly selected 80% of them as the training set and 20% as the test set.

#### Results

The results are shown in Table 18.

**Table 18:** Results for NER task

Model	BERT	XLNet	RoBERTa	ALBERT	Ensemble learning
Acc	0.938	0.951	0.886	0.894	0.964

#### Discussion

First, we use the dataset to train each single classifier. We then use the trained model to make predictions on the test set data. We have four single classifiers, so there are four sets of prediction results. Then we evaluate the performance of the model and get the accuracy of each classifier. Next, we use the accuracy rate as the weight of the classification result of a single model. Finally, the results of our ensemble model are obtained through weight voting.

### Using the ensemble learning method for text generation task

In this section, we discuss in detail our ensemble learning model for the text generation task.

#### Customized architecture

In the text generation task, the ensemble learning method we adopted is the same as that used in the NER task, and we still use the weighted voting method. First, we test the individual classifiers and observe their performance as individual classifiers. Then we use the accuracy of the classifier on the test set as the weight in the weighted voting method to vote on the result.

#### Hyperparameters

The detailed hyperparameters are shown in Table 19.

**Table 19:** The weight of different classifiers

Model	BERT	XLNet	GPT2	RoBERTa	ALBERT
Weight	0.316	0.111	0.362	0.265	0.417

#### Dataset

The data we used to validate the model’s text generation capabilities came from [53].

#### Results

Through the first row of Table 20, we find that the accuracy of using the weighted voting method to integrate the five classifiers is slightly lower than the best-performing single classifier. So, we decided to remove the single classifier with the lowest accuracy from the ensemble model each time to observe the performance of the ensemble model. The reason for this is to achieve a better classification effect through the integration of several high-quality single classifiers.

**Table 20:** Results for different combination

Ensemble model	Acc
XLNet+Albert+BERT+GPT2+RoBERTa	0.407
Albert+BERT+GPT2+RoBERTa	0.409
BERT+GPT2+RoBERTa	0.409
GPT2+RoBERTa	0.417
RoBERTa	0.417

#### Discussion

From the performance of the ensemble learning models combining different classifiers, we can see that the performance of the ensemble learning method is slightly lower than the best single classifier. The possible reasons for this result are as follows. First of all, RoBERTa, which has the best classification performance, has already covered all the correct classification results during classification. Therefore, when using the ensemble learning model of weight voting, the classification result is always equal to or lower than the performance of the RoBERTa classifier. Secondly, the reason for the general performance of the ensemble learning model may also be that the model only uses simple linear superposition, without more complicated operation logic. This resulted in the performance of the ensemble learning model being slightly lower than the best single classifier.

The comparison table is shown in Table 21. The comparison table lists the different transformer-based models used in the study along with the downstream tasks we were tested on, the datasets used, and the advantages and limitations of each model. The table also includes a column for comparing each model with others in the study.

**Table 21:** Comparison table

Name of the model/method	Downstream tasks	Downstream datasets	Advantages and limitations of the model/method	Comparison with other models/methods
BERT [3]	Sentiment analysis, question answering, NER, text summarization, topic modeling, text generation	Coronavirus tweets dataset, SQuAD 1.1, Groningen Meaning Bank corpus, CNN daily mail dataset, Disaster tweets dataset, Trump 2020 election speech dataset	Bidirectional approach for better contextual understanding.	Outperforms traditional models like LSTM and Gated Recurrent Unit (GRU). Outperforms GP2 in classification tasks.
ALBERT [7]	Sentiment analysis, question answering, NER, topic modeling, text generation	Coronavirus tweets dataset, SQuAD 1.1, Groningen Meaning Bank corpus, Disaster tweets dataset, Trump 2020 election speech dataset	Improved efficiency and reduced parameter count. Can be trained on relatively small batch sizes.	Achieves similar results to BERT with fewer parameters and smaller batch sizes.
RoBERTa [6]	Sentiment analysis, question answering, NER, text summarization, topic modeling, text generation	Coronavirus tweets dataset, SQuAD 1.1, Groningen Meaning Bank corpus, CNN daily mail dataset, Disaster tweets dataset, Trump 2020 election speech dataset	Training with dynamic masking for better generalization. Computationally expensive	Outperforms in many benchmarks
XLNet [5]	Sentiment analysis, question answering, NER, topic modeling, text generation	Coronavirus tweets dataset, SQuAD 1.1, Groningen Meaning Bank corpus, Disaster tweets dataset, Trump 2020 election speech dataset	Overcomes limitations of sequential pre-training.Requires longer training times than BERT	Outperforms BERT and ALBERT in some benchmarks
GPT2 [4]	Sentiment analysis, question answering, topic modeling, text generation	Coronavirus tweets dataset, SQuAD 1.1, Disaster tweets dataset, Trump 2020 election speech dataset	Pre-trained on large corpus of text for generalization	Achieves high performance in language generation tasks
Our proposed ensemble learning models	Sentiment analysis, NER, text generation	Coronavirus tweets dataset, Groningen Meaning Bank corpus, Trump 2020 election speech dataset	Combines strengths of multiple models for better accuracy. Requires additional computation for inference	Outperforms individual models in sentiment analysis and NER tasks

### Applicability range of ensemble methods in NLP tasks

In NLP, the effectiveness of ensemble methods is not a one-size-fits-all proposition. It varies across tasks due to a multitude of factors. Here, we highlight the situations where ensemble learning may prove beneficial or potentially less advantageous.

In certain tasks like sentiment analysis or NER, ensemble methods often provide significant advantages. These tasks typically benefit from the integration of diverse models, each offering unique perspectives on the data. An ensemble method can aggregate the strengths of the different models, thus improving overall performance. As observed in our research, the deep neural network ensemble method combined the outputs of various models for sentiment analysis and NER tasks, leading to improved accuracy and F1 scores.

However, the utility of ensemble methods may be reduced in some cases. Text generation tasks, especially those not involving downstream task training, might exemplify such a case. Here, a single well-performing model could potentially outperform an ensemble of models. This is because the task might favor a model with a particular set of features or training methodology. For instance, a model that has been pre-trained on a large corpus and employs an unsupervised learning approach might be more suitable.

In such a situation, if one model’s performance is significantly superior to the others, ensemble methods might be limited by the best classifier’s performance. If the high-performing model is already capturing the majority of the correct predictions, combining it with other less effective models might not result in a substantial performance increase.

Moreover, ensemble methods might not offer added value if the participating models are very similar or highly correlated in their predictions. In these instances, combining the models might increase the computational complexity without necessarily enhancing the performance.

A careful consideration of computational resources is also important when deciding between ensemble methods and single models. Ensemble methods often require more computational power and longer training times. If computational resources are restricted, using a single well-tuned model might be a more practical choice.

In summary, the decision to utilize ensemble methods should be informed by a deep understanding of the task at hand, the specific strengths and limitations of the models under consideration, and the available computational resources. Future research could aim to explore more sophisticated ensemble strategies and their applicability to a wider array of NLP tasks.

## Gap analysis and next steps

In this section, we conducted a gap analysis and decided on the tasks for the next stage.

### Gap analysis

1. While we evaluated several natural language processing models, we acknowledge that there are many other models that could have been used for the tasks we performed. Future work should consider a more comprehensive evaluation of various models to determine the optimal approach for each task.
2. Our experiments focused exclusively on methods based on the transformer model, and we did not compare our results to those obtained using other machine learning or deep learning methods. Future work should explore a wider range of approaches to determine the most effective method for each task.
3. Although we proposed an ensemble method for text generation, our results indicate that its performance was not particularly good. This may be due to the limitations of the ensemble learning method we used. Our proposed method relied on the output of a single model rather than considering model structure. Future work should investigate alternative ensemble learning methods that incorporate multiple models more effectively.
4. We did not use ensemble learning methods for the tasks of question answering, text summarization, and topic modeling. Future work should consider the use of ensemble learning methods to improve the performance of these tasks.

### Next steps

1. In the follow-up research, first we will expand the scope of natural language processing tasks and conduct research in more tasks. Secondly, we will expand the use of models and use more models in different tasks to make the direct comparison of models more comprehensive.
2. Subsequently, we will also use machine learning-based methods and deep learning methods to complete natural language processing tasks. Moreover, when using ensemble learning methods, we will not only consider models based on the transformer model but also integrate machine learning methods and some other deep learning methods. This can effectively integrate the advantages of different models and obtain a better-performing classification method.
3. For ensemble learning models, we will use more ensemble methods to integrate different classifiers. And we will consider integrating the structures of the models. After proposing a variety of ensemble models and conducting experimental comparisons, we may potentially find the best solution for each of the natural language processing tasks.
4. We also want to use and implement the ensemble learning methods on question answering, text summarization, and topic modeling tasks through the integration of the internal structures of the models. Through the integration of the internal structure, some of the above-mentioned challenges could be overcome to achieve better performance.

## Conclusions

In this research paper, we delve into the area of natural language processing, employing five distinct well-known transformer-based models to solve six different natural language processing tasks. Our primary focus revolves around analyzing the strengths and weaknesses of these models, and drawing insightful comparisons based on the results.

Our main contribution is that we carefully study the performance of these models on different natural language processing tasks. Given the variance in the performance of the models, we perform an extenstive analysis of all the models' structural nuances and training methods. A comprehensive analysis of different models on different tasks allows us to uncover the underlying factors that lead to performance differences between these models.

After identifying the unique advantages offered by the different models, we develop ensemble learning models using the ensemble learning methods to leverage their strengths in different aspects. The first ensemble learning model involves pooling the outputs of the individual model classifiers into a deep neural network, thereby achieving an ensemble learning effect. The second ensemble learning model leverages the accuracy of the individual models as weighted votes in a voting model, following an alternative well-known approach to ensemble learning. Through the analysis of the results of some specific natural language processing tasks, our ensemble learning models have better performance compared to the performance of a single classifier.

## Data Availability

Not applicable.

## Abbreviations

NLP: Natural language processing
LSTM: Long short-term memory
NER: Named entity recognition
SVM: Support vector machine
CRF: Conditional random field
RNN: Recurrent neural network
BERT: Bidirectional Encoder Representations from Transformers
ALBERT: A Lite BERT for Self-supervised Learning of Language Representations
RoBERTa: A Robustly Optimized BERT Pretraining Approach
